# Multifactorial stroma-mediated resistance is a major contributor to residual disease under targeted therapies in lung cancers

**DOI:** 10.21203/rs.3.rs-6264377/v1

**Published:** 2025-04-24

**Authors:** Bina Desai, Tatiana Miti, Sandhya Prabhakaran, Daria Miroshnychenko, Menkara Henry, Viktoriya Marusyk, Pragya Kumar, Hilal Ozakinci, Chandler Gatenbee, Marilyn Bui, Theresa A. Boyle, Jacob Scott, Philipp M. Altrock, Eric Haura, Alexander R.A. Anderson, David Basanta, Andriy Marusyk

**Affiliations:** 1 Department of Tumor Microenvironment and Metastasis, H Lee Moffitt Cancer Centre and Research Institute; Tampa, FL, USA; 2 Cancer Biology Ph.D. Program, University of South Florida; Tampa, FL, USA; 3 Department of Integrated Mathematical Oncology, H Lee Moffitt Cancer Center and Research Institute; Tampa, FL, USA; 4 Department of Thoracic Oncology, H. Lee Moffitt Cancer Center and Research Institute; Tampa, FL, USA; 4 Department of Pathology, H. Lee Moffitt Cancer Center and Research Institute; Tampa, FL, USA; 5 Department of Machine Learning, H. Lee Moffitt Cancer Center and Research Institute; Tampa, FL, USA; 7 Department of Translational Hematology and Oncology Research, Cleveland Clinic; Cleveland, OH, USA; 8 Department of Theoretical Biology, Max Planck Institute for Evolutionary Biology; Ploen, Germany; 9 Department of Molecular Medicine, University of South Florida; Tampa, FL, USA

## Abstract

Despite inducing strong and durable clinical responses, targeted therapies do not eliminate advanced cancers, as a subset of tumor cells survives within residual tumors, eventually developing resistance. The ability of tumor cells to avoid therapeutic elimination can be mediated both by cell-intrinsic and microenvironmental mechanisms. Whilst the specific molecular mediators of cell-intrinsic and microenvironmental resistance are well understood, their relative contribution to *in vivo* therapeutic responses remains poorly defined. Using spatial histological inferences from experimental models of ALK+ NSCLC, we found that peristromal niches protected tumor cells from therapeutic elimination in vivo, enabling in vivo persistence. Whereas the development of bona fide resistance is associated with the development of the development of cell-intrinsic resistance, relapse of tumor growth reflects a combined effect of both cell-intrinsic and microenvironmental mechanisms. Mechanistically, the protective effect of the peristromal niche is not reducible to a single mechanism, instead reflecting a combined effect of multiple juxtacrine and paracrine mediators. The lack of reducibility to a single molecular mediator presents an obvious challenge to the therapeutic paradigms of targeting individual resistance mechanisms. We found that this challenge could be mitigated by shifting the therapeutic focus to orthogonal collateral sensitivities of residual tumors. Exploiting adaptive upregulation of HER2, associated with both cell-intrinsic and microenvironmental persistence, using the antibody-drug conjugate T-DXd strongly enhanced the effect of targeted therapies and suppressed the development of resistance.

## One Sentence Summary

Peristromal niches protect lung cancer cells from therapeutic elimination and contribute to relapse through a combination of multiple molecular mechanisms.

## INTRODUCTION

Therapies that target oncogenic signaling addictions can induce strong and durable remissions in cancers defined by relevant mutational drivers. However, a subset of tumor cells typically persists under therapy, eventually giving rise to resistance. The main effort in the quest to solve the challenge of the inevitable failure of targeted therapies has been directed against the identification and targeting of the molecular mechanisms that underlie cancer survival (persistence) and resistance (positive net growth). This effort has led to the uncovering of a large and growing number of molecular mechanisms, including mutational changes in therapeutic targets, mutational or non-mutational activation of bypass signaling, histological transformation, and others (1). However, apart from the improvements that stem from overcoming the effects of common on-target mutations through improved inhibitor design, our knowledge of resistance mechanisms has not translated into therapeutic advances.

To a large extent, this paucity in therapeutic advances stems from intra-tumor heterogeneity of resistance mechanisms (2). When subpopulations with different resistance mechanisms are present at relapse, suppression or elimination of the dominant subpopulation cannot be expected to yield more than a temporary effect. A less appreciated but likely equally important issue is that, even at the level of individual tumor cells, therapeutic resistance can result from a combined effect of multiple mechanisms (3–5). Subsequently, suppressing one of the mechanisms within a compound resistance scenario does not lead to a full re-sensitization. Finally, therapy resistance is not limited to cell-intrinsic sources. A large body of experimental evidence points to contribution from the tumor microenvironment (TME), as paracrine and juxtacrine factors produced by tumor stroma are known to confer therapy resistance to otherwise sensitive tumor cells (6–8). These effects, which we describe under the umbrella term of stroma-mediated (SM) resistance (SMR), have been well-documented across various therapeutic contexts but appear to be particularly relevant for targeted therapies directed against oncogenic signaling addictions, such as inhibitors of EGFR and ALK mutations in non-small cell lung cancers (NSCLC). While specific cases of experimental or clinical relapse are typically attributed to a single dominant mechanism, either cell-intrinsic or stroma-mediated, this attribution is likely an oversimplification. Common experimental models of targeted therapy sensitivity are typically capable of developing therapy resistance under stroma-free *in vitro* culture conditions (3,5,9–11). Yet, the same experimental models can be rendered partially or fully therapy-resistant by co-culture with either cancer-associated fibroblasts (CAFs) (the main cellular component of TME), CAF conditioned media (CM), or specific CAF-produced cytokines, extracellular matrix (ECM) components, and other CAF-produced factors (8,12,13). Therefore, *in vivo*, therapeutic responses could be expected to reflect a combined contribution of both cell-intrinsic resistance and SMR mechanisms.

Uncovering the relative contribution of cell-intrinsic resistance and SMR to *in vivo* therapeutic responses could be potentially achieved by disabling one of them and examining the resulting effect on therapeutic responses in experimental animal models. Whereas cell-intrinsic therapy resistance is typically attributed to a single mutational or non-mutational mechanism, our studies with experimental models of ALK+ NSCLC revealed a more complex scenario of multifactorial resistance (5). Subsequent work of Franca et al. indicated a wide generalizability of this multifactorial underpinning, proposing the concept of a “resistance continuum”(3). Since the multifactorial underpinning of cell-intrinsic resistance limits its experimental disabling, we decided to focus on identifying and suppressing the molecular mediator of SMR. Given the major role of CAFs in mediating SMR across different contexts of targeted therapies (14–16), we reasoned that an experimental uncoupling of cell-intrinsic resistance and SMR effects might be achievable by identifying and disabling the mechanism(s) responsible for the resistance-conferring effects of CAFs. However, we found that, even at the level of a single experimental model, *in vivo* SMR cannot be reduced to a single molecular mediator. Instead, it reflects a combined effect of multiple juxtacrine and paracrine mechanisms, whose relative contribution changes over the course of therapy. Using spatial analyses of histological samples, we found that SMR plays a dominant role in the ability of tumors to persist and avoid therapeutic elimination. Whereas the relative contribution of SMR diminishes with the transition from remission to the resumption of tumor growth, our analyses indicate that tumor relapse integrates the effects of cell-intrinsic resistance and SMR mechanisms. The complex, multifactorial underpinning of therapeutic responses poses a major challenge to the efforts to overcome therapy resistance. We found that shifting focus from individual resistance mechanisms towards orthogonal therapeutic sensitivities of tumor cells within residual disease and using combination therapies that utilize the bystander effects of antibody-drug conjugates offers a promising solution to the challenges faced by precision oncology.

## RESULTS

### Stromal fibroblasts reduce sensitivity of NSCLC cells to targeted therapies via HGF-cMET axis *in vitro*

To assess the contribution of SMR towards *in vivo* therapeutic responses, we chose to focus on a common experimental model of ALK+ NSCLC, H3122 cell line. ALK+ NSCLC to ALK can be viewed as a “poster child” of both the success and limitations of targeted therapies. While ALK inhibitors typically induce strong and often durable clinical responses, acquired resistance remains unavoidable in advanced metastatic ALK+ NSCLC. Similar to many other experimental models of different contexts of targeted therapies, H3122 cultures can persist under therapy, withstanding therapeutic elimination and eventually developing resistance through cell-intrinsic mechanisms (5). At the same time, ALKi sensitivity of H3122 cells can be greatly reduced in the presence of CAFs (15–19). To dissect the relative contribution of cell-intrinsic, stroma-mediated persistence and resistance to *in vivo* responses to ALKi, we sought to uncouple them by identifying molecular mediator(s) of SMR *in vitro* and examining the effect of its suppression *in vivo*.

**Fig. 1.**
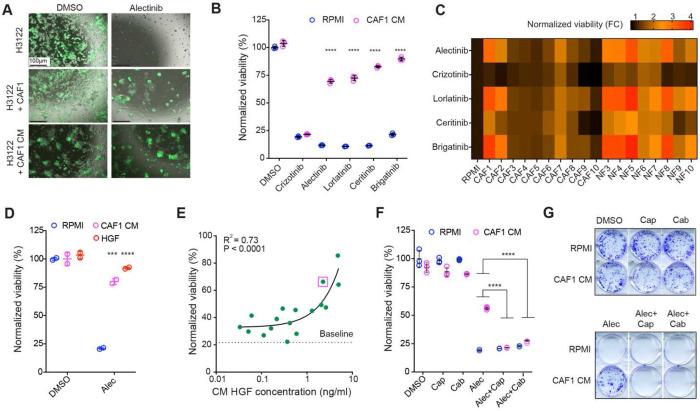
Fibroblasts protect ALK+ NSCLC from ALKi through the HGF-cMET axis. (**A)** Live fluorescent microscopy images of GFP labeled H3122 cells co-cultured with unlabeled CAFs or CAF1 CM in the presence of alectinib (0.5μM) or DMSO vehicle control. (**B**) Cell Titer-Glo viability assay of H3122 cells cultured in RPMI or CAF CM with DMSO control, crizotinib (0.5 μM), alectinib (0.25 μM), lorlatinib (0.5 μM), ceritinib (0.1 μM) and brigatinib (0.25 μM). Results are normalized to the DMSO control signal in RPMI. (**C**) Heatmap summary of the impact of fibroblast CM on sensitivity to H3122 cells to the indicated ALKi. The sensitivity is presented as the fold change in cell viability as compared to the RMPI media control. **(D**) Exogenous HGF at the concentration found in CAF1 CM (2 ng/ml) phenocopies alectinib-protecting effect of CAF1 CM. (**E**) Pearson correlation analysis of the correlation between HGF levels detected in the CM of individual fibroblast isolates and the impact of CM on alectinib (0.1 μM) sensitivity. Purple square highlights CAF1 CM. (**F, G**) cMET inhibitors capmatinib (0.2 μM) and cabozantinib (0.2 μM) overcome protective effect of CAF1 CM to alectinib (0.25 μM) in short-term (4d) viability assay (F) and longer-term clonogenic survival assay (**10d**), (**G**). *** and **** indicate p<0.001 and p<0.0001, respectively, of the interaction term of two-way ANOVA assay, comparing the impact of CM on viability of H3122 cells under treatment with ALKi versus vehicle control (DMSO) (B, D), or on viability of H3122 cells under alectinib in the absence or presence of cMET inhibitors (F).

Consistent with our previous work, co-culture with a primary, non-immortalized lung cancer CAF isolate (subsequently referred to as CAF1) (17) substantially reduced the sensitivity of H3122 to the frontline ALKi alectinib (Fig. 1A). CAF1 CM fully phenocopied the effect of a physical CAF1 co-culture, indicating that the protective effect is mediated by a secreted factor(s) (Fig. 1A). CAF1 CM conferred similarly strong protection to clinically approved ALK inhibitors lorlatinib, ceritinib and brigatinib, but not to crizotinib, which co-inhibits cMET (Fig. 1B). A similar effect was observed in a different model of ALK+ NSCLC, the STE-1 cell line (Fig. S1A). Indicative of the generalizability of these observations towards other targeted therapies in NSCLC, CAF1 CM conferred strong protection against EGFR inhibitors gefitinib and erlotinib in the PC9 cell line model of EGFR mutant NSCLC, capable of cell-intrinsic persistence and resistance (9,11,20) (Fig. S1B) as well as against KRAS^G12C^ inhibitors sotorasib and ARS-1620 in the H358 model of KRAS^G12C^ NSCLC (Fig. S1C). Next, given the known phenotypic diversity of fibroblasts(16,21), we examined the effect CM from a panel of primary human NSCLC CAF isolates as well as fibroblasts isolated from normal lung tissue (NF) (Fig. S1D). Despite a variability in the magnitude of the effect, CM from most fibroblast isolates protected H3122 cells against alectinib, lorlatinib, ceritinib, and brigatinib, while having little to no protection against crizotinib (Fig. 1C).

The lack of protection against crizotinib suggested mediation by the well-established bypass signaling mechanism, activation of cMET expressed by tumor cells by hepatocyte growth factor (HGF), produced by CAFs (15,18,22). Indeed, exogenous HGF conferred strong dose-dependent protection against alectinib in H3122 and STE-1 models (Fig. S2A, B). Exogenous HGF, added at the concentrations detected in CAF1 CM (2 ng/ml), fully phenocopied the protective effect of CAF1 CM against alectinib and lorlatinib (Fig. 1D, S2C), while providing no detectable protection against crizotinib (Fig. S2C). Importantly, we observed a strong correlation (R^2^=0.73) between HGF concentration in the CM of different fibroblast isolates and the magnitude of their protection for H3122 cells against alectinib (Fig 1E), indicating a generalizability of the CAF1 CM inferences toward other fibroblast isolates. Consistent with the central role of the HGF-cMET axis in mediating the effects of CAFs, the addition of an HGF-neutralizing antibody strongly suppressed the protective effects of CAF CM in H3122 and STE-1 models (Fig. S2D, E). Similarly, cMET inhibitors cabozantinib and capmatinib completely suppressed CAF-mediated protection in experimental models of ALK+, EGFR mutant, and KRAS^G12C^ NSCLC (Fig. 1F, S2F–I). Importantly, protection observed in short-term growth assays translated into longer-term protection in clonogenic assays (Fig. 1G, Fig. S2J). In summary, the substantial reduction of ALKi sensitivity of H3122 cells in the presence of fibroblast CM reflects a general phenomenon of CAF-mediated targeted therapy resistance. At least *in vitro*, the protective effect of CAFs on ALKi sensitivity in the H3122 model can be largely reduced to the well-characterized HGF-cMET bypass signaling mechanism.

### Modulation of the HGF-cMET axis moderately impacts alectinib responses *in vivo*

Assuming the mechanisms mediating the effect of CAFs are conserved between *in vitro* and *in vivo* contexts, the contribution of SMR to alectinib responses of H3122 tumors *in vivo* should be assessable by experimental manipulation of the HGF-cMET axis in xenograft models. However, murine HGF (mHGF) does not activate human cMET(23) (Fig. S3A), complicating the assessment and suggesting that SMR might be irrelevant for ALKi responses in conventional murine xenograft models.

**Fig. 2.**
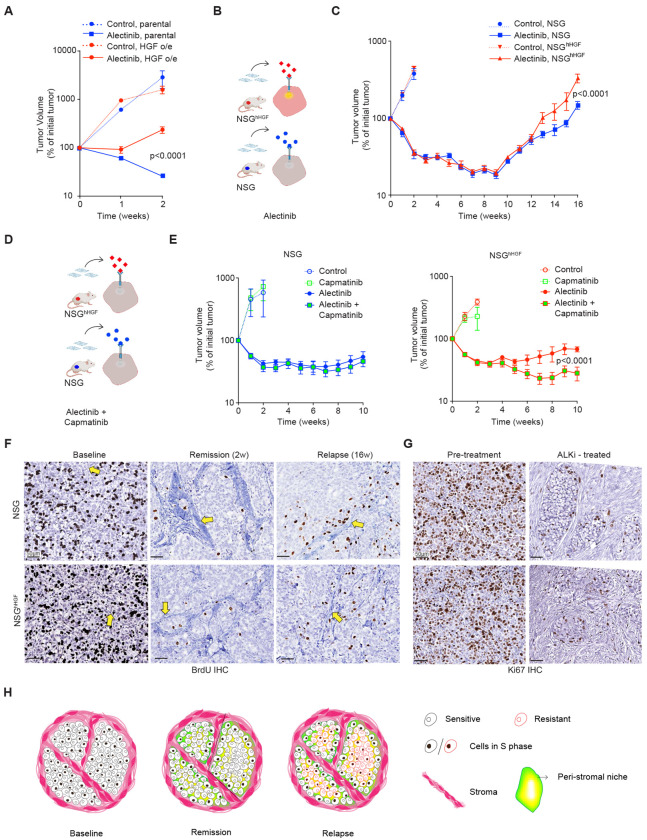
Stromal mediated resistance effects involve HGF independent mechanisms *in vivo*. (**A)** Exogenous expression of HGF confers resistance to alectinib (25 mg/kg) in NSG xenografts. N=4 tumors for all groups except for HGF o/e control (N=2). Error bars represent SEM. (**B**) Diagram of the experimental design to assess the impact of stromal activation of tumor cMET -mediated bypass signaling *in vivo*. (**C**) Volumetric response dynamics of H3122 xenograft tumors in NSG and NSG^hHGF^ hosts, continuously treated with 25 mg/kg alectinib or vehicle control. Error bars represent SEM; N= 4 & 18 for the control and alectinib-treated groups, respectively. (**D**) Diagram of the experimental idea: cMET inhibition should suppress the enhanced relapse in NSG^hHGF^ xenografts. (**E**) Tumor response dynamics for vehicle control (N=4), 40 mg/kg capmatinib (N=4), 20 mg/kg alectinib (N=10) and alectinib/capmatinib (N=10) combination treated groups respectively. (**F**) Representative images of anti-BrdU IHC from xenograft tumor tissues at the indicated time points. Arrows point to the examples of stromal regions defined by counterstain. P values in tumor growth analyses represents the significance of the interaction term of the repeated measurement 2-way ANOVA analyses between the indicated groups. (**G**) Representative images of anti-KI67 IHC tumor tissue from pre-treatment or post ALKi treatment of patient diagnosed with ALK+ NSCLC. H. Conceptual model for the contribution of SMR to the *in vivo* therapeutic responses

To overcome this limitation, we first evaluated the ability of human HGF (hHGF) to confer *bona fide* resistance *in vivo*. To this end, we overexpressed hHGF in H3122 cells using a lentiviral vector and compared alectinib responses between NSG xenograft tumors formed by subcutaneous grafts of H3122 parental cells, lacking detectable HGF expression and their hHGF overexpressing counterparts. Whereas hHGF expression had no discernable impact on the baseline tumor growth, it prevented tumor regression in response to alectinib (Fig. 2A, S3B), indicating that the HGF-cMET axis represents a strong resistance mechanism. However, the observation of the autocrine hHGF-mediated resistance does not directly address the question of the impact of the stroma-produced HGF, as the paracrine-acting effects of SMR mediators might be spatially limited (24). Therefore, to evaluate the impact of the HGF-conferred SMR more directly, we sought to leverage the full compatibility between human HGF and murine cMET; a replacement of both murine HGF alleles in the NSG mice with their human counterparts produces no detectable phenotype, apart from modulating responses of hHGF-responsive human xenografts (25). We reasoned that contrasting therapy responsiveness of parental H3122 cells grafted in the regular NSG strain versus its humanized derivate (NSG^hHGF^) should provide a “clean” test of the impact of SMR on therapeutic responses (Fig. 2B).

Consistent with the lack of impact of HGF on the baseline proliferation of H3122 cells both *in vitro* (Fig. 1D) and *in vivo* (Fig. 2A), we observed no discernable differences in tumor implantation efficiency and baseline tumor growth (Fig. 2C, S3C, D). Surprisingly, initial tumor responses to alectinib were indistinguishable between the two mouse models. A moderate reduction of alectinib sensitivity in tumors grafted in NSG^hHGF^ mice was only detectable at relapse (Fig. 2C, S3D, E). To further characterize the impact of the HGF-cMET axis on *in vivo* alectinib sensitivities, we assessed the ability of a highly specific cMET inhibitor, capmatinib, to abrogate the effect of hHGF. (Fig. 2D). Given the relatively modest differences between the two models, we used a slightly lower alectinib dosing (20 mg/kg instead of 25 mg/kg) to enhance the ability to detect the effect of the HGF-cMET axis modulation. Consistent with the inability of mHGF to activate human cMET, capmatinib had no discernable effect on the alectinib response of H3122 tumors grafted into NSG mice. In contrast, in NSG^hHGF^ H3122 xenografts, capmatinib substantially enhanced the magnitude of tumor regression and delayed the relapse (Fig. 2E, S3F, G), supporting the relevance of the HGF-cMET axis for stroma-mediated resistance *in vivo*.

### Proximity to stroma reduces alectinib sensitivity *in vivo* through a combination of HGF-dependent and independent mechanisms

Given the strong effect of ectopic HGF expression *in vivo* (Fig. 2A) and the ability of fibroblast-produced HGF to provide ALKi protection *in vitro* (Fig. 1), we reasoned that the modest effect of stromal hHGF in xenograft models could reflect the spatial limitations, as cMET activation can be expected to be limited to tumor cells in the immediate proximity to hHGF-producing stroma. To address this possibility, we examined the spatial distribution of tumor cell proliferation in the two xenograft models, using IHC staining of tumor tissue for BrdU, which marks cells undergoing DNA replication. Without treatment, the distribution of BrdU+ cells in tumors collected from animals pulsed with BrdU prior to euthanasia appeared to be random in both the NSG and NSG^hHGF^ xenograft tumors (Fig. 2F, baseline). Consistent with the therapy-protecting effects of CAFs *in vitro*, while alectinib treatment potently suppressed cell proliferation, BrdU+ cells could still be observed in the peristromal niches (Fig. 2F, remission). Unexpectedly, this effect was detected in both xenograft tumor models, indicating the action of HGF-independent SMR mechanism(s) that overshadow the effects of the HGF-cMET axis (Fig. 2F). Tumor relapse was associated with a partial restoration of tumor cell proliferation. Consistent with the reduction in cell-intrinsic ALKi sensitivity, the bias in cell proliferation toward peristromal niches was diminished. However, it has not completely disappeared (Fig. 2F, relapse), indicating that the tumor relapse is driven by a combination of both cell-intrinsic and SMR effects.

The bias in tumor cell proliferation toward peristromal niches under ALKi therapy was not limited to alectinib treatment, as lorlatinib induced a similar effect in H3122 tumors grafted in regular NSG mice that lacked the functional HGF-cMET axis (Fig. S4A). Moreover, the effect was observed in additional experimental models of ALK+ NSCLC. Similar to the H3122 tumors, in the absence of therapy, the distribution of BrdU+ cells appeared random in the NSG xenografts of STE-1, H2228, and MGH006-1 tumors (Fig. S4B–D). Alectinib treatment strongly suppressed proliferation, inducing bias in the distribution of BrdU+ cells towards the ECM-rich stroma (Fig. S4B–D), suggesting the broad generalizability of the SMR. Importantly, IHC staining for a common proliferation marker Ki67 revealed a similar ALKi-induced stroma proximity bias in cell proliferation in clinical samples (Fig. 2G, Fig. S5). These observations suggest the following conceptual model (Fig. 2H): suppression of oncogenic addiction by targeting therapies suppresses proliferation and induces cell death. However, intrinsically therapy-sensitive cells within peristromal niches are partially protected from these effects. Thus, SMR might represent the major source of in vivo persistence. The SMR protected cells eventually develop partial cell-intrinsic resistance, enabling their proliferation and survival outside of the peristromal niche. However, their proliferation might still be boosted by paracrine signals produced by stroma.

**Fig. 3.**
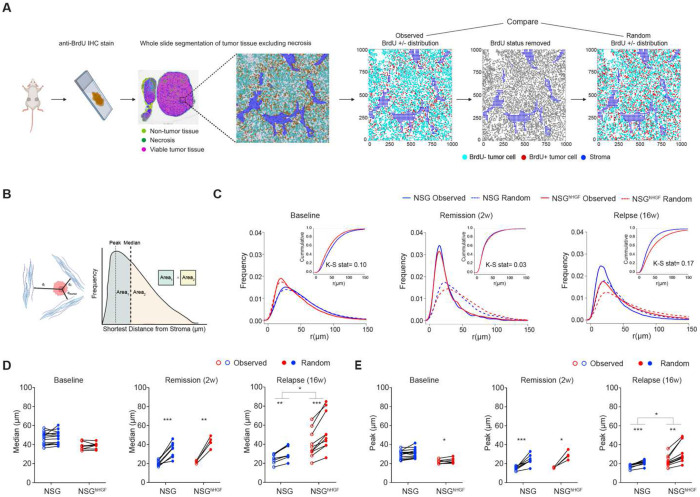
Spatial analyses of tumor tissues indicate HGF-independent sheltering from cytostatic effects of alectinib in peristromal niches. (**A)** Diagram of the spatial analysis workflow. **(B)** Diagram depicting analyses of distances to the nearest stroma. **(C)** Observed and random distributions of the distances of BrdU+ cells to the nearest stroma; cumulative distribution and KS statistics values are shown as insets. (**D, E**) Ladder plots comparing the medians (D) and means (E) of predicted and observed distributions of the distances of BrdU+ cells to the nearest stroma. Each point reflects an analysis of a whole cross-section of an individual tumor. *, **, *** indicate p values of less than 0.05, 0.01, and 0.001, respectively. Comparisons between the observed and predicted values were performed using a 2-tailed t-test; comparisons between NSG and NSG^hHGF^ models were performed with the analyses of the interaction term of 2-way ANOVA test. KS denotes Kolmogorov-Smirnov test.

To quantitatively assess the impact of stroma proximity on tumor cell proliferation and to evaluate the relative contribution of the HGF-cMET axis, we took advantage of our digital pathology/spatial analysis pipeline (26). In this approach, scanned microscopy images of the anti-BrdU IHC-stained histological tumor cross-sections are segmented into necrotic areas (excluded from the analyses), stromal regions, BrdU+, and BrdU− tumor cells. Continuous stromal regions, defined by clearly distinguishable ECM deposition, are further rasterized to facilitate quantitation (Fig. 3A).

Our ability to draw accurate quantitative inferences from spatial distributions requires adequate controls. Since therapy reduces the tumor/stroma ratio and alters the relative distribution of tumor parenchyma and stroma, direct comparisons between the control and alectinib-treated tumors are not easily interpretable. To overcome this limitation, the spatial distributions of BrdU+ tumor cells were compared to their sample-specific digital “avatars”. These digital avatars retain specific spatial locations of each tumor cell and stromal regions, but the BrdU positivity status is randomly redistributed among the tumor cells while preserving the BrdU+ fraction (Fig. 3A). Comparison between the observed and predicted random distributions enables accurate assessment of the spatial proliferation bias in each sample. Comparisons of pairwise (observed versus predicted random) differences between vehicle control and alectinib-treated groups enable the assessment of the impact of therapy. Finally, comparisons of the predicted/observed differences between the NSG and NSG^hHGF^ xenograft tumors enable the assessment of the impact of stromal HGF. Importantly, our pipeline analyses all tumor cells within the histological cross-sections (80K-400K per tumor), with multiple independent tumors analyzed per time point, thus minimizing the impacts of spatial heterogeneity and sampling biases.

To assess the impact of stroma proximity on cell proliferation, we assessed the distributions of distances of BrdU+ tumor cells to the nearest stroma (24,27) (Fig. 3B), with the predicted random distributions serving as sample-specific null hypothesis controls. Our analyses confirmed the visual inferences of the lack of spatial bias in NSG and NSG^hHGF^ xenograft tumors in the absence of therapy (Fig. 3C–E). In contrast, we detected a strong spatial bias in BrdU+ tumor cells towards peri-stromal regions in alectinib-treated tumors. While this bias was most pronounced in regressing tumors, it was also present upon relapse (Fig. 3C–E). Consistent with the lack of detectable HGF-dependence on the initial tumor responses (Fig. 2C), we did not detect differences in the stroma proximity bias between the NSG and NSG^hHGF^ tumors during remission. However, at relapse, the bias was significantly stronger in the NSG^hHGF^ xenografts (Fig. 3C–E), consistent with the divergence of volumetric responses (Fig.2C). Taken together, our spatial analyses support the notion that stroma-mediated resistance contributes to both incomplete tumor responses and tumor growth relapse, and indicate that, *in vivo*, stroma-mediated resistance cannot be reduced to the effects of the HGF-cMET axis alone.

**Figure 4.**
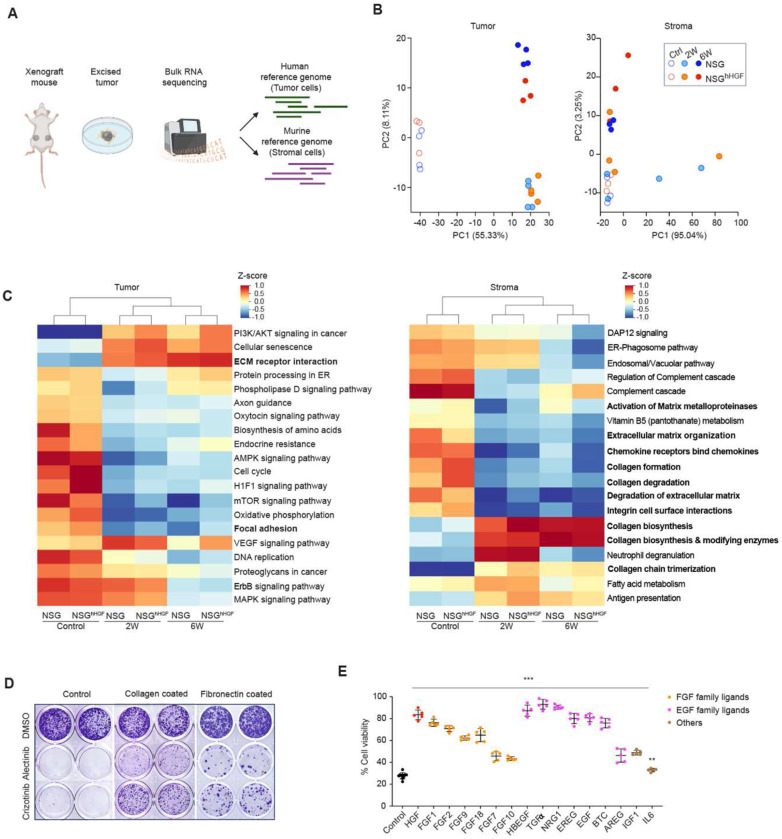
Transcriptomic analyses of tumor and stromal components identify the potential HGF-independent mechanisms of stroma-mediated therapy resistance. **(A**) Diagram for transcriptional analyses approach. (**B**) PCA analyses of neoplastic and stromal transcriptomes. (**C**) Non-supervised hierarchical clustering of pathway analyses of differentially expressed genes. Bolded font indicates pathways related to ECM production and remodeling. (**D**) Crystal violet stain of clonogenic assay of H3122 cells seeded into control, collagen or fibronectin coated plates cultured for 20 days in 0.1 μM alectinib, 0.5 μM crizotinib or DMSO control. (**E**) Cell Titer Glo viability assay for H3122 cells cultured in 0.1 μM alectinib in the presence of indicated growth factors (50ng/ml). ** and *** indicate p<0.01 and p<0.001 respectively for the 2-tailed Mann Whitney test comparing alectinib sensitivity between the control samples and samples treated with the indicated growth factors

To uncover the identity of the HGF-independent SMR mechanism(s), we performed transcriptomic analyses of treatment-naïve and alectinib-treated (2 and 6 weeks) tumors from the H3122 NSG and H3122 NSG^hHGF^ xenograft models using bulk Illumina RNA sequencing. Reads were mapped to both human and murine genomes, enabling simultaneous assessment of the neoplastic and stromal compartments (Fig. 4A). Principle component analysis (PCA) and nonsupervised hierarchical clustering of the neoplastic transcriptomes clearly distinguished samples from treatment naïve and alectinib-treated tumors at different treatment time points (Fig. 4B, Fig S6A). Consistent with the lack of the baseline and early response differences between the NSG^hHGF^ and NSG xenografts in tumor growth and spatial distributions of cell proliferation, transcriptional analyses revealed the emergence of the HGF-dependent differences only after six weeks of treatment (Fig. 4B, Fig. S6A). Pathway enrichment analyses indicated therapy-induced, HGF-independent suppression of multiple pathways related to cell cycle, metabolism, and signaling, as well as therapy-induced activation of PI3-AKT signaling, cellular senescence, and ECM-receptor interactions (Fig 4C). To assess the utility of transcriptional inferences in identifying potential therapeutic vulnerabilities, we assessed the impact of suppressing PI3K signaling, one of the pathways differentially expressed between therapy-naïve and alectinib-treated tumors with PI3K inhibitor alpelisib. Consistent with a recent report (28), alpelisib treatment significantly enhanced tumor responses of xenograft tumors to alectinib, supporting the utility of the transcriptomics inferences in identifying pathways mediating alectinib resistance (Fig. S6B).

PCA and hierarchical clustering analyses of transcriptional reads mapped to the mouse genome revealed a clear impact of alectinib treatment on global transcriptomes. However, the effects of the treatment duration and stromal expression of human HGF were less pronounced (Fig. 4B, Fig. S6A). Pathway enrichment analyses revealed marked changes in multiple pathways involved in ECM synthesis, degradation, and organization in alectinib-treated tumors (Fig. 4C). Consistent with these changes, Mason Trichrome staining of histological samples revealed a therapy-induced enhancement of collagen deposition (Fig. S6C). Additionally, IHC staining with hyaluronan-binding protein revealed a substantial increase in the deposition of hyaluronan, which was previously implied in microenvironmental therapy resistance (24) (29) (Fig. S6D).

The ECM-tumor cell interaction was dispensable for the ability of CAFs to reduce alectinib sensitivity in our *in vitro* studies, as CM was capable of phenocopying the effects of CAF co-cultures (Fig. 1A). However, we reasoned that this might be a reflection of specific experimental conditions, as stronger resistance in physical CAF co-cultures was documented under higher cell density and CAF/H3122 ratios (30). Integrin-mediated cell adhesion to collagens and fibronectin is a well-established juxtacrine SMR mechanism documented in multiple contexts of targeted therapies (31,32). Murine stroma expressed multiple ECM components implied in microenvironmental resistance, while their expression was undetectable in human neoplastic cells (Fig. S6E). To assess the potential contribution of collagens and fibronectin on the therapeutic sensitivity of ALK+ NSCLC to ALKi, we contrasted the responses of H3122 and STE-1 cells grown on regular tissue culture plastic with those grown on collagen-coated or fibronectin-coated plates. We found that cells grown on the collagen and fibronectin matrix were substantially less sensitive to both alectinib and crizotinib (Fig. 4D, S7A), supporting the potential involvement of cell-ECM interactions in HGF-independent mediation of SMR to ALKi.

Next, we evaluated the potential involvement of paracrine-acting cytokines and growth factors previously implied in microenvironment-mediated resistance to targeted therapies (8,12,13). Transcriptional analyses revealed that similar to the genes encoding for ECM components, expression of these growth factor genes was primarily confined to the stroma (Fig. S6E). While not all the potential mediators exerted a detectable effect on alectinib sensitivity *in vitro*, multiple EGF and FGF family growth factors as well as IGF1 and IL6, substantially reduced the sensitivity of H3122 cells to alectinib in short-term growth assays and clonogenic studies (Fig. 4E, Fig S7B, C), supporting their potential involvement in mediating the HGF-independent SMR effects *in vivo*, where high local concentrations might be achieved in peristromal niches, compared to the dilutions in the CAF CM. In summary, our analyses suggest that similar to the previously reported multifactorial nature of cell-intrinsic therapy resistance (3,5), SMR might reflect an integrated action of multiple juxtacrine and paracrine mechanisms.

### Targeting individual mechanisms is insufficient to overcome the effect of SMR.

**Figure 5.**
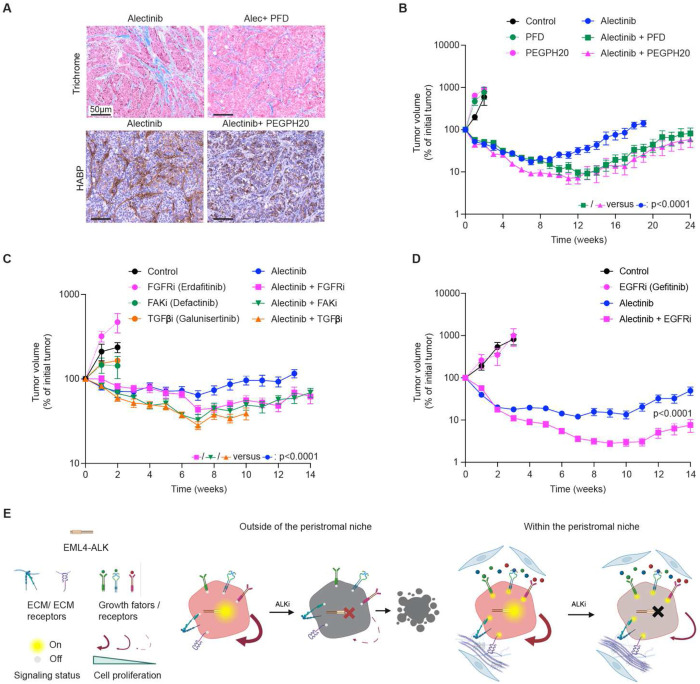
Suppression of multiple candidate mechanisms of stromal resistance improves responses to ALKi. (**A**) Pirfenidone and PEGPH20 treatment suppresses alectinib-induced enhancement of collagen (Mason Trichrome staining, upper panel) and hyaluronan (lower panel) deposition, respectively. (**B)** Volumetric responses of H3122 xenograft tumors to vehicle (N=4), 900 mg/kg pirfenidone (N=6), 0.1 mg/kg PEGPH20 (N=6), 25 mg/kg alectinib (N=10), alectinib/PEGPH20 and alectinib/pirfenidone combinations (N=10). (**C**) Volumetric responses of H3122 xenograft tumors to vehicle, 20 mg/kg erdafitinib, 50 mg/kg defactinib, 75 mg/kg galunisertib, 20 mg/kg alectinib and the indicated combinations. N=6 for the control and individual co-inhibitors. N=10 for the alectinib and the combination treatment groups. (**D**) Volumetric responses of H3122 xenograft tumors to vehicle, 20 mg/kg alectinib, 40 mg/kg gefitinib and alectinib/gefitinib combination. N=10 for the alectinib and combination treatment groups, N=6 for the control and gefitinib group. P values indicate the result of the interaction group of repeated measurement ANOVA comparing combination therapies to the alectinib monotherapy. (**E**) Diagram of the conceptual model. Peristromal niche location enables tumor cells to partially maintain viability and proliferation upon ALK inhibition due to multiple ECM and growth factor signaling mechanisms. Outside of the peristromal niche, cells lack the compensating mechanisms, leading to a more complete proliferation arrest and apoptosis.

To assess the contribution of the HGF-independent SMR *in vivo*, we evaluated the impact of pirfenidone on the alectinib sensitivity of H3122 tumors implanted in regular NSG mice. Pirfenidone is a well-established anti-fibrotic agent that reduces fibroblast activation that acts by suppressing the enhanced production of multiple ECM components and cytokines (33,34), thus simultaneously suppressing multiple putative mechanisms that can mediate the HGF-independent SMR. As expected, pirfenidone reduced the alectinib-induced increase in the deposition of collagens in H3122 xenografts (Fig. 5A). While pirfenidone treatment did not impact tumor growth, it substantially enhanced both the magnitude and the duration of alectinib-induced remission (Fig. 5B, S8A). Given that the anti-fibrotic effects of pirfenidone are mediated by the suppression of TGF-β production (33,34), we assessed the effects of an independent modulator of TGFb responses using galunisertib, an inhibitor of TGF-β receptor I. Similar to pirfenidone, galunisertib enhanced alectinib responses (Fig. 5C, S8B). Importantly, TGF-β has no direct effect on reducing the sensitivity of H3122 cells to alectinib (Fig. S7B), enabling a straightforward assessment of tumor cell-extrinsic effects. In summary, these results support the notion of a substantial contribution of HGF-independent SMR mechanisms to *in vivo* therapeutic responses to ALKi.

Next, we narrowed our analyses to assessing the contribution of more specific candidate mechanisms of SMR. Hyaluronan, a common ECM component, is known to facilitate EM resistance to EGFR/HER2 inhibitor lapatinib in breast cancers (24). Thus, we evaluated the effect of the PEGylated recombinant human hyaluronidase (PEGPH20) (35), which substantially reduced alectinib-induced hyaluronan deposition (Fig. 5A). Similar to pirfenidone, PEGPH20 co-treatment substantially enhanced alectinib sensitivity of H3122 tumors (Fig. 5B, S8A). A similar enhancement of alectinib sensitivity was observed with a co-inhibition of focal adhesion kinase (FAK), which integrates juxtacrine effects of integrin-mediated adhesion to collagens and fibronectins (Fig. 5C, S8B). To evaluate the relevance of HGF-independent paracrine mechanisms, we assessed the contributions of FGF and EGF-mediated signaling by suppressing their receptors. Consistent with our *in vitro* data (Fig. 4E, S7C), FGFR inhibitors erdafitinib and infigratinib, and EGFR inhibitor gefitinib, enhanced alectinib sensitivity, delaying tumor remissions (Fig. 5C, D, S8B–D). Notably, histological analyses of cell proliferation revealed that none of the inhibitors of individual SMR mechanisms was able to suppress the proliferation of tumor cells within peristromal niches completely (Fig. S8E), supporting the notion of the multifactorial underpinning of the SMR (Fig. 5E).

### Targeting collateral sensitivities bypasses SMR.

**Figure 6.**
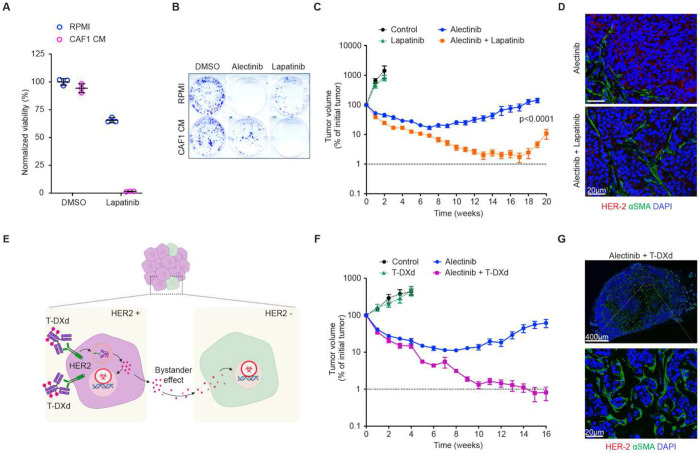
Targeting collateral sensitivity associated with stromal proximity overcomes the limitations of multifactorial resistance. (**A)** Impact of CAF1 CM on sensitivity of H3122 cells to 10 μM lapatinib in 4 days CellTiter-Glo assay. (**B)** Impact of CAF1 CM on sensitivity of H3122 cells to 0.1 μM alectinib and 10 μM lapatinib in 10 days Crystal Violet clonogenic assay. (**C**) Response dynamics of H3122 xenograft tumors treated with vehicle control (N=5), 100 mg/kg lapatinib (N=6), 25 mg/kg alectinib (N=10) and alectinib/lapatinib combination (N=10). Error bars represent SEM. (**D**) Representative images of IF staining against HER-2 (red) and αSMA (green) of xenograft tumors from (C) at the experimental endpoint. (**E**) Diagram of the experimental idea: targeting of HER2 expressing cells with the antibody-drug conjugate T-DXd should lead to elimination of the rare HER2- cells via bystander effect. (**F**) Response dynamics of H3122 xenograft tumors treated with vehicle control (N=4), 10 mg/kg T-DXd (N=8), 25 mg/kg alectinib (N=8) and alectinib/T-DXd combination (N=10). (**G)** Representative images of whole tumor against HER-2 (red) and αSMA (green) of xenograft tumors collected at the endpoint of the experiment shown in (F).

The combined contribution of multifactorial cell-intrinsic resistance and SMR to therapeutic responses poses an obvious limitation to the success of the common strategy of identifying and targeting a single persistence/resistance mechanism at a time. On the other hand, the feasibility of simultaneous targeting of multiple mechanisms of cell-intrinsic and cell-extrinsic resistance is limited by the iatrogenic challenge of additive toxicity. Potentially, this limitation can be addressed by shifting therapeutic focus from targeting specific persistence/resistance mechanisms towards targeting orthogonal collateral sensitivities of the residual tumors. Our previous work has revealed that active transcriptional reprogramming during the acquisition of resistance to alectinib is associated with the emergence of a strong collateral sensitivity to a dual EGFR/HER2 inhibitor lapatinib, enabling to sidestep the challenge posed by multifactorial cell-intrinsic resistance (5). Given the parallels between SMR and cell-intrinsic persistence, we assessed the impact of CAF CM on lapatinib sensitivity. CAF1 CM exposure dramatically enhanced the lapatinib sensitivity of H3122 and STE-1 cells in both short-term and clonogenic growth assays (Fig. 6A, B and Fig. S9A, B). This effect was observed with CM from multiple fibroblast isolates (Fig. S9C–E) and, unlike cell-intrinsic collateral sensitivity(5), was not dependent on alectinib pre-treatment.

Expression of both EGFR and HER2 protein was detectable in H3122 xenograft tumors at baseline (Fig. S9F, G), and was further enhanced after two weeks of alectinib treatment, especially for HER2 (Fig. S9F). Consistent with this observation and our previous report(5), lapatinib treatment dramatically enhanced alectinib responses in H3122 xenografts, inducing stronger tumor regression compared to the enhancement of individual SMR mechanisms (Fig. 6C and Fig. S10A). However, the lapatinib-alectinib combination failed to eradicate H3122 tumors and suppress their ability to develop resistance and relapse. Examination of relapsed tumors indicated that most of the tumor cells have lost HER2 expression (Fig. 6D and Fig. S10B), suggesting a competitive release of HER2-independent subpopulations or an acquired loss of HER2 dependence.

We reasoned that this therapeutic escape could be mitigated using a HER2-targeting antibody-drug conjugate (ADC) trastuzumab-deruxtecan (T-DXd) in lieu of the small molecule inhibitor lapatinib. Anti-tumor effects of trastuzumab do not rely on the cell’s dependence on HER2 signaling, and the bystander effects of the T-DXd payload, topoisomerase inhibitor deruxtecan enable targeting rare subpopulations that do not express HER2 (Fig. 6E). T-DXd strongly enhanced the alectinib responses in H3122 xenograft tumors, driving 6/10 tumors below the volumetric detectability threshold (Fig. 6F and Fig. S10C). Histological examination of the injection sites revealed the presence of small pockets of residual disease composed of tumor cells that lacked detectable HER2 expression (Fig.S10D). A strong enhancement of alectinib responses was also observed in the STE-1 xenograft model. STE-1 xenograft tumors show stronger alectinib sensitivity compared to H3122, and within six weeks of treatment, multiple tumors within both the alectinib (4/10) and alectinib-T-DXd (8/10) treated groups regressed below volumetric detectability threshold, complicating the comparison (Fig. S10E). To overcome this limitation, we discontinued the treatment, focusing on the relapse dynamics. Whereas discontinuation of alectinib treatment led to an immediate relapse, volumetrically detectable tumors post alectinib-T-DXd treatment continued the regression, consistent with the lasting suppression of tumor cell growth associated with cytotoxic chemotherapies (26). Eventually, all of the tumors became undetectable, even after 11 weeks post discontinuation of treatment. Examination of the injection sites for evidence of residual disease revealed that only two out of ten sites contained small pockets of tumor cells. As with H3122 xenografts, residual STE-1 cells lost the detectable HER2 expression (Fig. S10F). Notably, the small pockets of HER2- STE-1 cells were completely encapsulated by hypertrophied fibrotic stromal tissue (Fig. S10F), suggesting a potential involvement of stromal reaction in suppressing tumor growth relapse. Importantly, the switch to the alectinib-T-DXd combination was able to restore therapeutic responsiveness in the tumors that have relapsed on alectinib monotherapy, indicating its utility in reversing the acquired therapy resistance (Fig. S10G, H). In summary, our results suggest that the challenge of the mechanistic complexity of persistence and targeted therapies can be mitigated by shifting the focus from identifying and targeting individual resistance-mediating pathways towards targeting collateral sensitivities and using therapeutic agents with broader therapeutic effects.

## DISCUSSION

Our study indicates that cancer’s ability to avoid eradication by targeted therapies and to develop resistance *in vivo* reflects a combined contribution of both cell-intrinsic resistance and SMR mechanisms. SM effects dominate *in vivo* persistence (residual disease) while also contributing to resistance (relapsed tumor growth). Importantly, our study indicates that, similar to the mechanistic complexity of cell-intrinsic resistance (3,5), SMR represents a multifactorial, compound phenotype that cannot necessarily be reducible to a single dominant mechanism. Together with a generally recognized challenge of intra-tumor heterogeneity (2), this mechanistic complexity poses an obvious challenge to the common therapeutic strategies of identifying and targeting a single molecular mechanism. Our results suggest that this challenge can be partially mitigated by shifting therapeutic focus towards exploiting collateral sensitivities of tumor cells within the residual disease.

A large and growing body of experimental studies has demonstrated the ability of CAFs and other non-neoplastic cell types within tumor stroma to reduce targeted therapy sensitivity in a wide range of therapeutic contexts, particularly for targeted therapies (36). Mechanistically, these effects have been attributed to multiple stroma-produced paracrine factors, including cytokines, ECM components, metabolites, and non-coding RNAs within exosomes (36,37). Despite the multiplicity of documented mechanisms, individual studies typically ascribe the SMR to a single dominant mechanism per experimental system or tumor. Guided by the assumption of reducibility of CAF-mediated resistance to a single mechanism, we identified HGF-cMET signaling as a pathway predominantly responsible for CAF-mediated resistance *in vitro* (Fig. 1). However, our *in vivo* studies revealed a more complex scenario, where the SMR involves the combined contribution of several growth factors (HGF, FGF and EGF family ligands) and ECM components (integrins, fibronectin and hyaluronic acid). Importantly, our study did not exhaust the assessment of all of the potential SMR mechanisms uncovered in previous mechanistic studies, such as metabolic crosstalk (37) and extracellular vehicle-mediated miRNA transfer(38). Therefore, SMR is likely to be even more mechanistically complex, *e.g.*, including the contributions of additional molecular mediators.

Notably, our results suggest that the relative contribution of different mechanisms might change over the course of therapy. For example, whereas suppression of integrin signaling through a FAKi defactinib and targeting hyaluronidase through PEGPH20 immediately enhanced alectinib sensitivity (Fig. 5B, C), the effect of suppression of HGF-cMET and FGF-FGFR signaling could only be detected after a significant delay (Fig. 2C, E; Fig. 5C). These differences might reflect dynamic changes in both tumor and stromal microenvironment over the course of therapy, which might not be reducible to a clean sequence of sensitivity-persistence-resistance, instead developing over an eco-evolutionary resistance continuum, where the relative contribution of different mechanisms changes over the course of the adaptation. Alternatively, the lack of detectable effects of the HGF and FGF-mediated paracrine mechanisms during the earlier stages of response might indicate that at the initial stages of adaptation, the survival and proliferation of tumor cells might require a combined effect of both paracrine and juxtacrine mechanisms and thus can be only detected within the immediate proximity to the stroma. Over the course of the treatment, carcinoma cells within residual tumors develop partial cell-intrinsic resistance, which might enable them to survive and proliferate beyond the immediate proximity to the stroma. However, the relatively weak paracrine signals provide an additional fitness boost, enhancing tumor cell proliferation beyond the immediate proximity to stroma, required for the juxtacrine signaling (Fig2H).

Given the spatial limitations of SMR action, knowledge of its molecular mechanisms is insufficient for understanding its effects. The discrepancy between *in vitro* and *in vivo* findings highlights the importance of spatial microenvironmental heterogeneity. Juxtacrine interactions with CAF-produced ECM only impact tumor cells that are immediately adjacent to stroma, while multiple cytokines capable of reducing therapy sensitivity to targeted therapies are likely to reach sufficiently high concentrations only within the peristromal niche while being diluted in the CM. Thus, adequate understanding of the SMR effects requires consideration of the characteristic lengths scale of the paracrine action, stroma/tumor ratios, and spatial distribution of the protective stroma niches. While the methodology to capture these effects has yet to be fully developed, adequate quantitative inferences can be obtained through spatial analyses of histological images and further adapting metrics from the fields of spatial statistics and spatial ecology (39–42) (Fig. 3). However, histological analyses only capture a single temporal snapshot. Given the temporal dimension of the acquisition of therapy resistance uncovered in recent studies (5,10,43), an adequate understanding of the impact of SMR calls for consideration of both spatial and also temporal dynamics. This high complexity necessitates integrating mathematical modeling tools like spatial agent-based models(44,45) or compartmentalized dynamical systems(44).

The contribution of SM mechanisms to in vivo therapy resistance and the mechanistic complexity of SMR adds to the challenges posed by the diversity of resistance mechanisms, intratumor heterogeneity(2) and the multifactorial nature of cell-intrinsic therapy resistance identified in recent studies(5,10,43). While the development of new therapies relies on reductionistic molecular oncology studies, the ability of tumor cells to resist therapeutic elimination and acquired therapy resistance represents a complex dynamical phenomenon, integrating the impact of multiple cell-intrinsic mechanisms, tumor heterogeneity, and systemic factors (such as inflammation(46) and variability in drug concentrations due to pharmacokinetics modifiers(47)). Consequently, the current paradigm of identifying and targeting a single mechanism at a time is unlikely to bring major clinical breakthroughs. On the one hand, targeting individual mechanisms mediating SMR or cell-intrinsic resistance can enhance the effect of the primary therapeutic agent (Fig. 5B–D). On the other hand, co-targeting any resistance mechanism in isolation is insufficient to prevent the ability of neoplastic populations to survive and adapt. At the same time, simultaneous co-targeting of multiple resistance mechanisms is complicated by the issue of added toxicities and the moving target nature of the tumors under treatment.

While our study examined a limited set of experimental models, the majority of well-studied experimental models of targeted therapy responses are capable of persistence and evolved resistance yet can also benefit from therapy-protecting interactions mediated by stroma-produced ECM or growth factors. Of note, a complex interplay between cell-intrinsic resistance and SMR has been recently reported for the development of therapy resistance in bone metastatic lesions of multiple myeloma(48). Even though clinical analyses typically focus on cell-intrinsic resistance mechanisms, a higher stroma/tumor ratio strongly correlates with poor therapy and survival outcomes across multiple types of cancers, including NSCLC (49). This suggests the strong and widespread contribution of SMR effects, a notion that is further supported by the observation of therapy-induced spatial bias towards peristromal niches in clinical cancers (Fig. 2G, Fig. S5).

Our study highlights the potential utility of focusing on collateral vulnerabilities associated with therapy persistence. At least in the H3122 and STE-1 experimental models, both adaptive phenotypic rewiring during the acquisition of resistance to ALKi exposure to CAF produced secreted factors lead to strong enhancement in sensitivity to the dual EGFR/HER2 inhibitor lapatinib(5) (Fig. 6B, Fig. S9A–E). While we do not fully understand the underpinning of this shared sensitivity, it might reflect a common denominator of partial EMT, associated with enhanced phenotypic plasticity (37,50,51). Targeting this collateral sensitivity produces superior responses compared to targeting individual molecular mediators of SMR (compare Fig. 6C with Fig. 5C, D). However, the alectinib-lapatinib combination failed to eradicate the tumors and to prevent them from adapting, likely reflecting the existence of HER2-independent therapeutic escape mechanisms. This failure prompted us to consider the use of the antibody-drug conjugate T-DXd which combines target specificity with an on-tumor bystander effect offered by antibody-drug conjugates (Fig. 6E). While a subset of tumor cells still escaped eradication, the alectinib-T-DXd combination induced very strong responses, and the residual tumors were unable to re-grow after treatment discontinuation (Fig. S10E). Of note, a similar strong enhancement of tumor responses to the primary targeted therapy by T-DXd was observed in a panel of models of KRAS^G12C^ NSCLC (52), indicating a broad generalizability of our observations. In summary, explicit consideration of the mechanistic complexity of resistance opens the door to the orthogonal therapeutic strategies that mitigate this challenge.

## MATERIALS AND METHODS

### Cell culture

NSCLC cell lines H3122, STE-1, PC9, and H358 were obtained from the Lung Cancer Center of Excellence Cell Line depository at Moffitt Cancer Center. HGF, GFP and mCherry-expressing variants of H3122 cells were obtained as described(53). Cancer cell lines were cultured in RPMI (Gibco, ThermoFisher) supplemented with 10% fetal bovine serum (FBS), 1% penicillin/streptomycin, and 10μg/ml human recombinant insulin (Gibco) at 37°C and 5% CO_2_. All cell lines have been authenticated by short tandem repeat analysis.

Primary fibroblasts were derived from lung cancer patients’ tumors or adjacent non-cancerous lung tissue and were cultured in FB media as previously described(24). All human tissues were collected using protocols approved by the H Lee Moffitt Cancer Center Institutional Review Board. All cancer cell lines and fibroblast isolates were routinely tested for mycoplasma contamination.

Fibroblast CM was generated by growing to ~ 80% confluency in FB media, at which point the media was removed, the media was replaced with 10% FBS RPMI after a PBS wash. CM was collected after 48 hours, filtered (0.2 μm), aliquoted, and either used immediately or stored at −80°C. HGF concentration in the CM was determined using the human HGF ELISA Kit (Sigma-Aldrich #RAB0212) following manufacturer protocol.

For collagen coating of tissue culture plates, rat tail collagen 1 was ordered from Thermo Fischer (catalog #A1048301). Collagen 1 was further diluted to achieve 0.5 mg/ml in 0.02N glacial acetic acid, plates were coated with the standard protocol as reported by the manufacturer. Plates were then immediately used to seed cells for the experiment. Fibronectin-coated plates were ordered from Stem Cell Technologies (catalog #100-1195).

### *In vitro* viability assays

For short-term cell viability assay, 4000 cells per well were seeded in either RPMI or a 1:1 ratio of RPMI: Fibroblast CM in a 96-well plate. Treatment was initiated the next day and lasted for 3 days. Cell viability was determined using CellTiterGlo reagent (Promega) following the manufacturer’s recommended protocol. The luminescence signal was determined using a GloMax luminometer (Promega). For the analysis, the luminescence signal from the empty wells was subtracted from each reading as the background, and % cell viability was determined relative to DMSO-treated cells cultured in RPMI.

For cell viability in the co-culture setting, nuclear GFP labeled H3122 tumor cells were seeded with unlabeled fibroblasts in a 1:1 ratio along with monoculture controls, to match the total number of cells in the co-culture setting. The live cell images were acquired with the IncuCyte live cell imaging system in the green fluoresce channel (software versions 2016A/B).

For low-density long-term colony formation assays, cells were seeded in either 6-well or 24-well plates. Treatment was initiated 24 hours post seeding and lasted for 10 to 20 days. At the end point, the cells were washed with cold 1X PBS and fixed with cold methanol. Tumor cell colonies were stained with crystal violet solution (0.5% crystal violet solution in 25% methanol), followed by water wash. Plates were allowed to dry overnight and then scanned to generate the images.

### Reagents and cytokines

Alectinib, Crizotinib, Lorlatinib, Ceritinib, and Cabozantinib, were purchased from Fisher Scientific. Capmatinib, Sotorasib, and ARS-1620 were purchased from SelleckChem. Stock solutions of Brigatinib, Gefitinib, and Erlotinib were received as a kind gift from Uwe Rix lab at H Lee Moffitt Cancer Center. All drugs were dissolved in DMSO, aliquoted, and stored at −20°C. For the mouse studies, pharmaceutical grade alectinib, alpelisib, pirfenidone and T-DXd were received from the Lung Cancer Center of Excellence at Moffitt Cancer Center. Infigratinib, Galunisertib and Lapatinib was purchased from AstaTech. Capmatinib and Erdafitinib were purchased from MedChemExpress. Defactinib was purchased from Selleckchem.

The human HGF Antibody was purchased from R&D systems (AB-294-NA); murine HGF recombinant protein was purchased from Sino Biological (50038-MNAH-20). EGF (GMP100-15), HB-EGF (100-47), BTC (100-50), EREG (100-04), TGFα (100-16A), AREG (100-55B), BMP4 (GMP120-05ET), BMP6 (120-06), BMP7 (120-03P), TGFβ2 (100-35B), FGF1 (100-17A), FGF2 (100-18C), FGF7 (100-19), FGF10 (100-26), FGF9 (100-23), FGF18 (100-28), IL6 (200-06), IL11 (200-11), Activin A (120-14P) as well as murine growth factors & cytokines - IL11 (220-11), IL6 (216-16), FGF1 (450-33A), FGF2 (450-33), FGF7 (450-60), FGF10 (450-61), FGF9 (450-30), BTC (315-21), AREG (315-36), EGF (315-09) were purchased from PeproTech. Human recombinant NRG1 was purchased from BioLegend. We reconstituted these growth factors & cytokines in sterile 0.1% bovine serum albumin/ phosphate-buffered saline (FGF1- 5mM sodium phosphate and FGF2 - 5mM sodium chloride) and were stored at −20°C.

### Animal studies

Xenograft studies were performed by subcutaneous bilateral implantation of 5E6 tumor cells/injection suspended 100 μl of 1: 1 RPMI / BME type 3 (R&D Systems #36-320-1002P). 4 to 6-week-old NOD-scid IL2Rgnull (NSG) or the NSG derivative NSG^hHGF^ (https://www.jax.org/strain/014553) recipient mice of both sexes. The animals were produced at the institute with breeders purchased from Jackson Laboratory. Treatment was initialized 3 weeks post implantation. Small molecule inhibitors were administered via daily oral gavages, 100 μl per dose. Alectinib and pirfenidone were dissolved in water. Capmatinib, Alpelisib, Erdafitinib, Defactinib, Galunisertinib and Lapatinib were suspended in MCT buffer (0.25% of methyl cellulose and 0.05% of Tween 80 in water). PEGPH20 was intravenously administered at 0.1 mg/kg twice a week. T-DXd was administered via intraperitoneal injections every 2 weeks.

Tumor diameters were recorded weekly using electronic calipers; tumor volumes were calculated assuming spherical-shaped tumors. 30-45 minutes prior to euthanasia, the animals were injected IP with 10 mg/ml BrdU dissolved in 1X PBS. The weights of excised tumors were recorded, and tumors were fixed in 10% formalin solution and/or snap-frozen (in liquid nitrogen) for further analyses. All the xenograft studies were performed per the approved procedures of IACUC protocol #IS00005557 of the H. Lee Moffitt Cancer Center. Animals were maintained under AAALAC-accredited specific pathogen-free housing vivarium and veterinary supervision following standard guidelines for temperature and humidity, with a 12-hour light/12-hour dark cycle.

### Histological analyses

Formalin-fixed paraffin-embedded (FFPE) xenograft tumors were cut into 5-μm sections and mounted on glass slides. FFPE sections were deparaffinized and rehydrated, followed by heat-induced antigen retrieval in citrate buffer (pH 6.2). For immune histochemical staining, slides were incubated with 3% hydrogen peroxide in methanol followed by 10% goat serum blocking solution in 1X PBS for 10 min at room temperature. For BrdU staining, slides were incubated with primary anti-BrdU antibody (Roche,1117037600, 1:100) for 1 hour followed by the incubation with an anti-mouse biotinylated antibody (Vector Labs, BA-9200, 1:100) for 45 mins. For the detection of hyaluronic acid, slides were incubated with 4μg/ml biotinylated HABP (Sigma-Aldrich, catalog #385911) for 2.5 hours at room temperature. Detection of staining was performed with Vectastain ABC peroxidase reagent (Vector Laboratories, PK-6100) following the recommended protocol, using DAB as a colorimetric substrate and hematoxylin counterstain. Tissue collagen was detected using Masson Trichrome stain kit (Thermo Fisher, NC9752152) following manufacturer protocol. Following the staining, covering glass was mounted using permount^™^ mounting media (Thermo Fisher, SP15-100). BrdU, HABP and Mason Trichrome stained sections were scanned with Aperio Scan Scope XT Slide Scanner (Leica).

For immunofluorescence staining, deparaffinized and rehydrated FFPE unstained tissue samples were heat incubated with antigen retrieval buffer (pH9 or pH6.2). Post one hour of incubation with blocking reagent (VectorLabs #S6000-100), tissues were incubated with primary antibodies against HER-2 (cell signaling, #4290T, 1:200) or EGFR (cell signaling, #4267, 1:50) and αSMA (Agilent, M085129, 1:100) overnight at +4C. Secondary antibody staining was performed using Alexa Fluor 488, goat anti-mouse IgG, (Invitrogen # A32723,1:1000), and Alexa Fluor 594, goat anti-rabbit IgG, (Invitrogen # A11012, 1:500) followed by nuclear staining with DAPI (Sigma-Aldrich #D9542) (1:10,000 in PBS) for 10 mins at room temperature. Prior to cover slip mounting, slides were treated with Vector Trueview autofluorescence quenching kit (VectorLabs, #SP-8400). VECTASHIELD (VectorLabs #H-1200) medium was used for slide mounting. Immunofluorescence images were acquired using PhenoCycler-Fusion system (Akoya Biosciences).

### Tissue segmentation and spatial statistics analyses

Tissue segmentation was performed using the AI-assisted digital pathology platform Aiforia version 5.2 as described in(54). Tissues were segmented into BrdU+/− tumor cells, stroma, and necrotic tissue. The stromal areas identified by counterstaining were segmented as polygons separating cellular and acellular-ECM components. Accuracy of tissue segmentation was vetted by American Board of Pathology certified pathologist.

Spatial statistics analyses were performed using R 4.1.2. Point patterns were extracted from the Aiforia-segmented histological images as described previously(54). Euclidian distance to the nearest stroma pixel was determined for each of BrdU+ tumor cell. Cumulative density functions of the distributions were used to perform comparison between BrdU+ and BrdU− tumor cells using the Kolgomorov-Smirnoff (KS) test. To account for the variability in tumor/stroma distribution, each sample was compared to an *in silico* random control, which retained spatial information on each of the tumor cell and stromal pixel, but BrdU+ status was randomly redistributed among tumor cells.

### RNA-seq analyses

Snap-frozen xenograft tumor tissues were immersed in liquid nitrogen and physically disrupted with a mortar pestle. Total RNA was isolated using TRIzol reagent (Invitrogen, 15596026) according to the manufacturer’s protocol. The concentration, quality, and integrity for total RNA was measured using NanoDrop and TapeStation system. cDNA library preparation and quality assessment were accomplished using Novogene’s library guidelines. NovaSeq 6000 PE150 was used as a sequencing platform. Reads were mapped to the human - GRCh38 reference genome or murine - GRCm38/mm10 reference genome. Normalized reads were obtained from DeSeq2, and principal component analysis (PCA) was performed for Hg38 and mm10, to study transcriptomic changes in human tumor cells and murine stromal component of the tumor respectively.

To compare gene expression levels across baseline, remission, and relapse, we selected a list of significant genes per sample, using the DESeq2 R package(55). We filtered genes that have a fold change > |2| and false discovery rate (FDR) < 0.05. A ranked gene list was then created based on the genes’ log2 fold change values. Next, we generated cluster maps for Hg38 and mm10 using these DEGs. The clusters so obtained on differential expression per sample indicate genes with similar expression patterns. After clustering, we calculated the z-scores for each row (each gene) and plot these instead of the log normalized expression values; this ensured the expression patterns that we want to visualize were highlighted and not confounded by the normalized expression values. Red color indicates genes with high expression levels, and blue color indicates genes with low expression levels. The cluster maps reinforce the presence of DEGs unique to each sample, and the potential effects of treatment on the DEGs.

Gene set enrichment analysis for the mouse and human mapped genes was performed, using the KEGG7 database(56) (www.kegg.jp) and RNAlysis software(57) to identify the biological pathways that are significantly associated with each sample, and to assess the influence of treatment. We considered KEGG terms with p-adj < 0.05 as significant enrichment. To visualize the differential expressions of KEGG pathways across samples, we generated pathway heatmaps using the NBBt-test R package(58).

### Statistical analyses

In this study, all statistical analyses were performed using either Prism 9 (GraphPad Software) or R statistical software (R Foundation for Statistical Computing). Details for the statistical tests are provided in the figure legends. When relevant, the figure present experimental means ± SD or mean ± SEM. P < 0.05 was considered as a significant.

## Supplementary Material

1

## Data Availability

All data are available in the main text or the supplementary materials.
