# Supplementary material for: Multifactorial stroma-mediated resistance is a major contributor to residual disease under targeted therapies in lung cancers
Source: Res Sq. 2025 Apr 24:rs.3.rs-6264377. Preprint. [Version 1] doi: 10.21203/rs.3.rs-6264377/v1 (PMC12045365; doi:10.21203/rs.3.rs-6264377/v1)
Supplement: 1 [file NIHPPrs6264377v1-supplement-1.pdf]

Figure S1

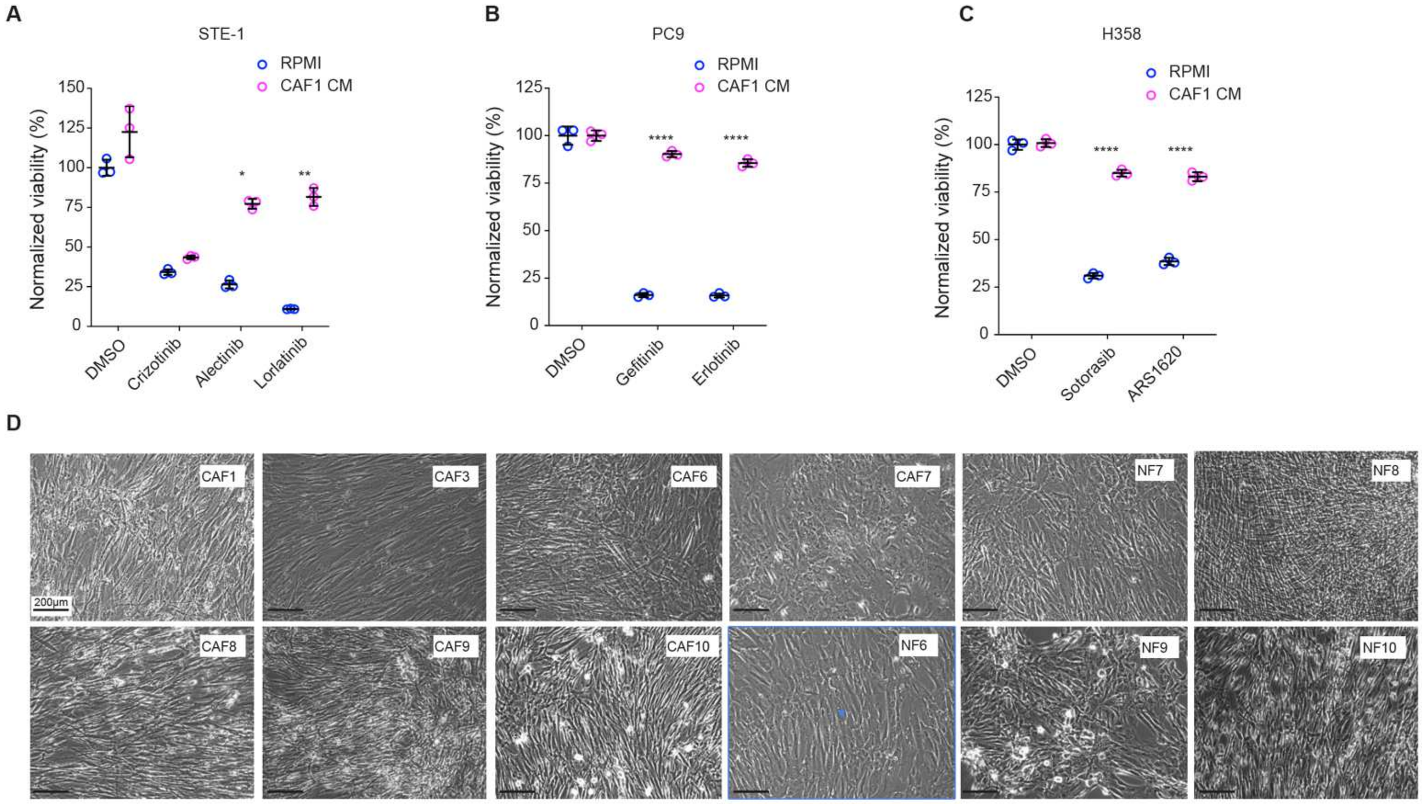

Figure S1. (**A)** Impact of CAF CM on the viability of STE1 cells in crizotinib (0.5 μM), alectinib (0.25 μM), lorlatinib (0.5 μM) and DMSO control. (**B**) Impact of CAF CM on the viability of PC9 cells in gefitinib (0.25 μM), erlotinib (0.25 μM) and DMSO control (**C**) Impact of CAF CM on the viability of H358 cells in sotorasib (0.05 μM), ARS1620 (1 μM) and DMSO control**.** *, **and **** refers to $p<0.05$, $p<0.01$ and $p<0.0001$, respectively, of the interaction term of two-way ANOVA assay, comparing the impact of CM on viability between DMSO and treatment groups. (**D**) Representative microscopy images of cultures of normal and cancer-associated fibroblasts, 10x magnification.

Figure S2

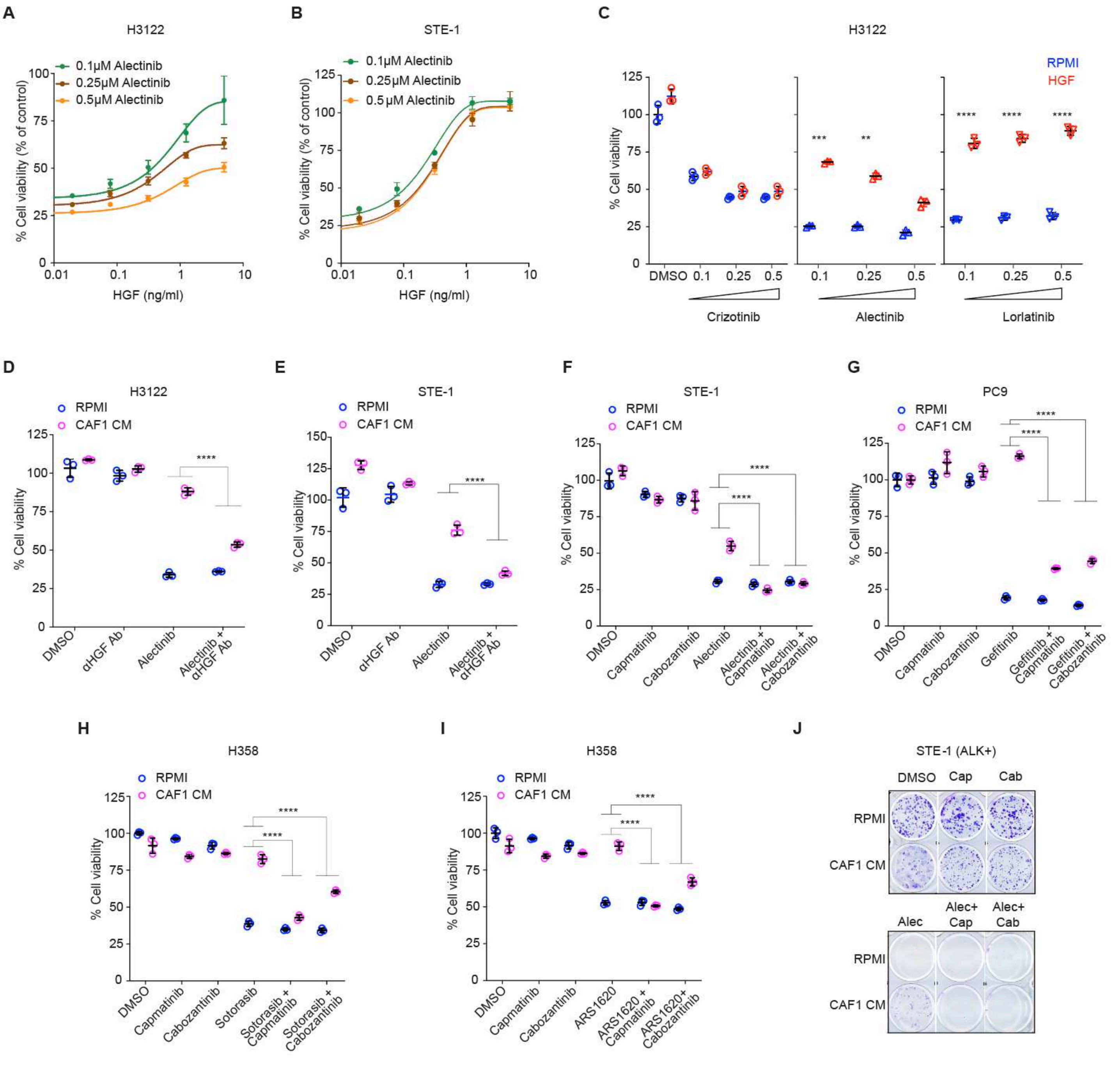

Figure S2. Effect of the indicated concentrations of HGF on the viability of H3122 (**A**) and STE1 (**B**) cells under the indicated alectinib concentrations. (**C**) Impact of HGF (2 ng/ml) on the viability of H3122 cells under the indicated concentrations of ALKi. (**D**, **E**) Impact of HGF neutralizing antibody (2.5 μg/ml) on the viability of H3122 (D) and STE1 (E) cells in 0.1 μM of alectinib. (**F-I**). Impact of cMET inhibitors cabozantinib (0.2 μM) and capmatinib (0.2 μM) on the sensitivity of STE-1 cells to 0.1 μM alectinib (F), PC9 cells to 0.25 μM gefitinib (G), H358 cells to 0.05 μM sotorasib (H) and 1 μM ARS1620 (I). **, ***, and **** indicate $p<0.01$, $p<0.001$, and $p<0.0001$ of the interaction term of 2-way ANOVA between the indicated groups. J. Microscopy images of the crystal violet stain of the 10-day culture of STE1 cells under 0.2 μM capmatinib, 0.2 μM cabozantinib, 0.1 μM alectinib, cMETi/alectinib combinations and DMSO control.

Figure S3

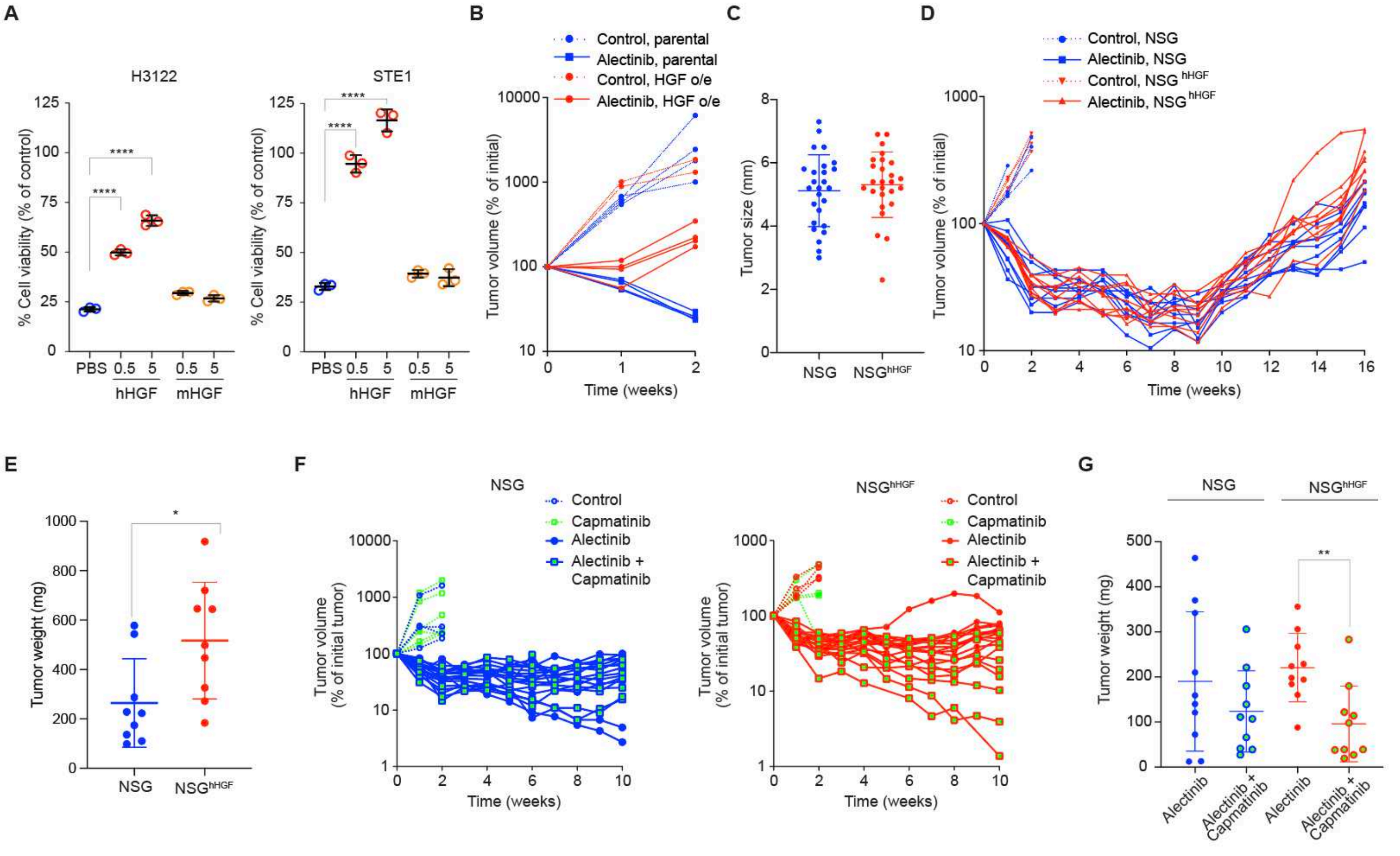

Figure S3. (**A**) Impact of the indicated concentrations of human and murine HGF on the viability of H3122 and STE1 cells under 0.1 μM alectinib. (**B**). Volumetric traces of individual parental and HGF expressing H3122 xenograft tumors treated with 25 mg/kg alectinib or vehicle control. (**C**) Tumor sizes (diameters) of xenograft tumors in NSG and NSG^{hHGF} hosts pre-treatment. Each dot represents an individual tumor. (**D**) Volumetric traces of individual H3122 xenograft tumors in NSG and NSG^{hHGF} hosts treated with 25 mg/kg alectinib or vehicle control. (**E**) Final tumor weights from the experiment depicted in (D). (**F**). Volumetric data of individual H3122 xenograft tumors treated with 40mg/kg capmatinib, 25 mg/kg alectinib or vehicle control in NSG (left panel) or NSG^{hHGF} (right panel) hosts. (**G**). Final tumor weights from the experiment depicted in (F).

Figure S4

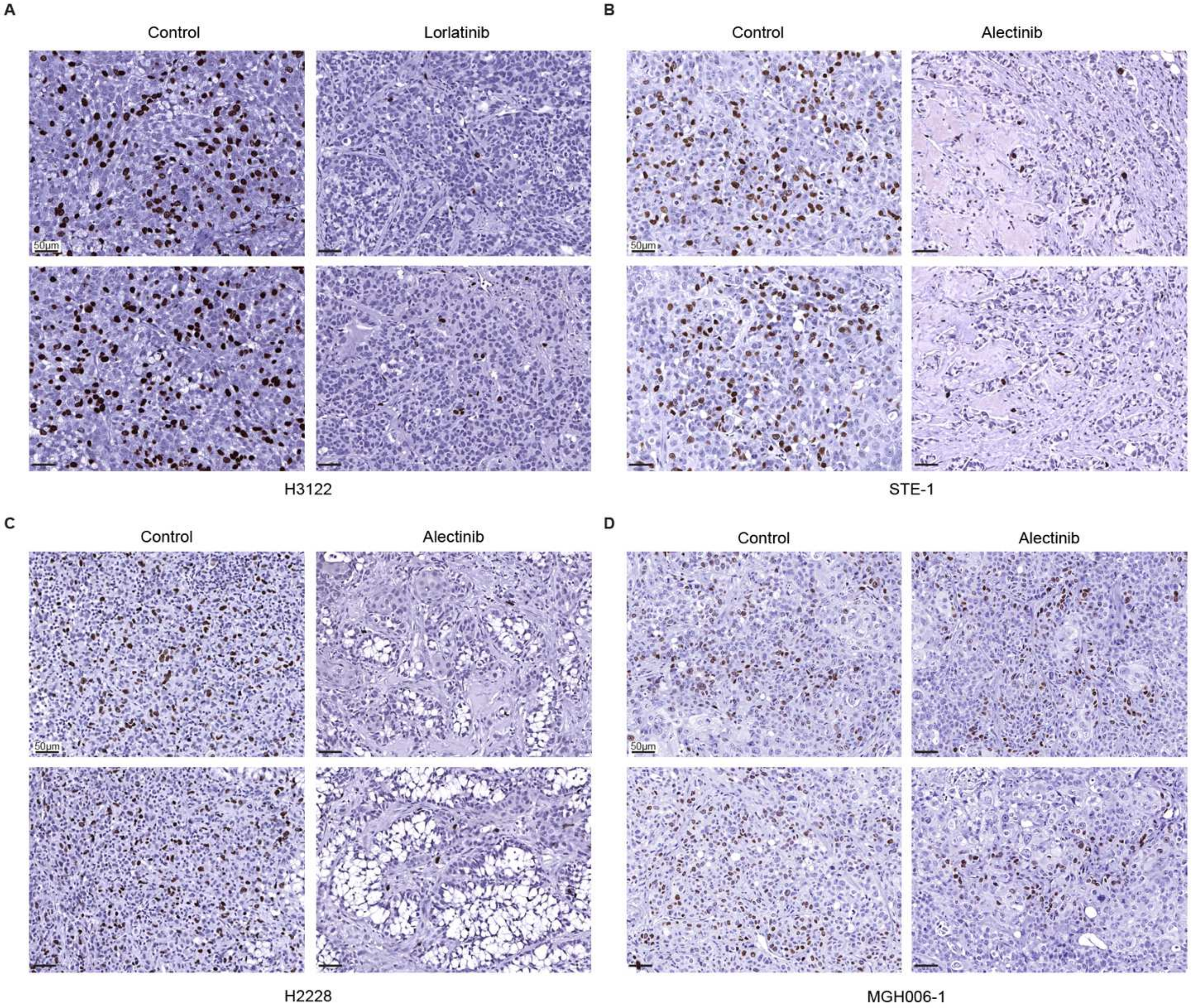

Figure S4. Representative images of anti-BrdU IHC staining of tumors tissues from H3122 (**A**), STE-1 (**B**), H2228 (**C**) and MGH006-1 (**D**) xenograft mice treated with the indicated therapies.

Figure S5

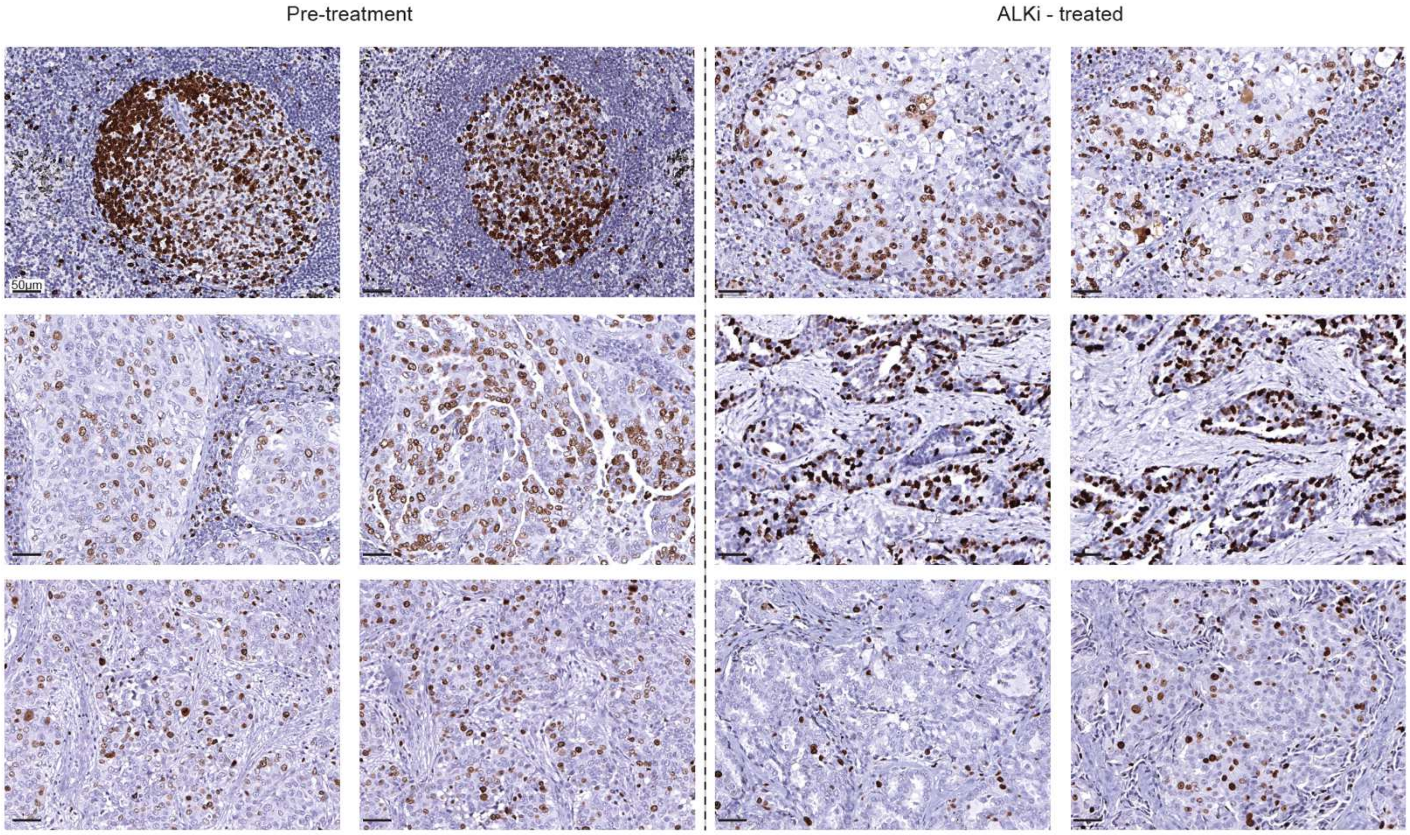

Figure S5. Representative images of anti-KI67 IHC tumor tissue from pre-treatment (left panel) or post ALKi treatment (right panel) of patient diagnosed with ALK+ NSCLC.

Figure S6

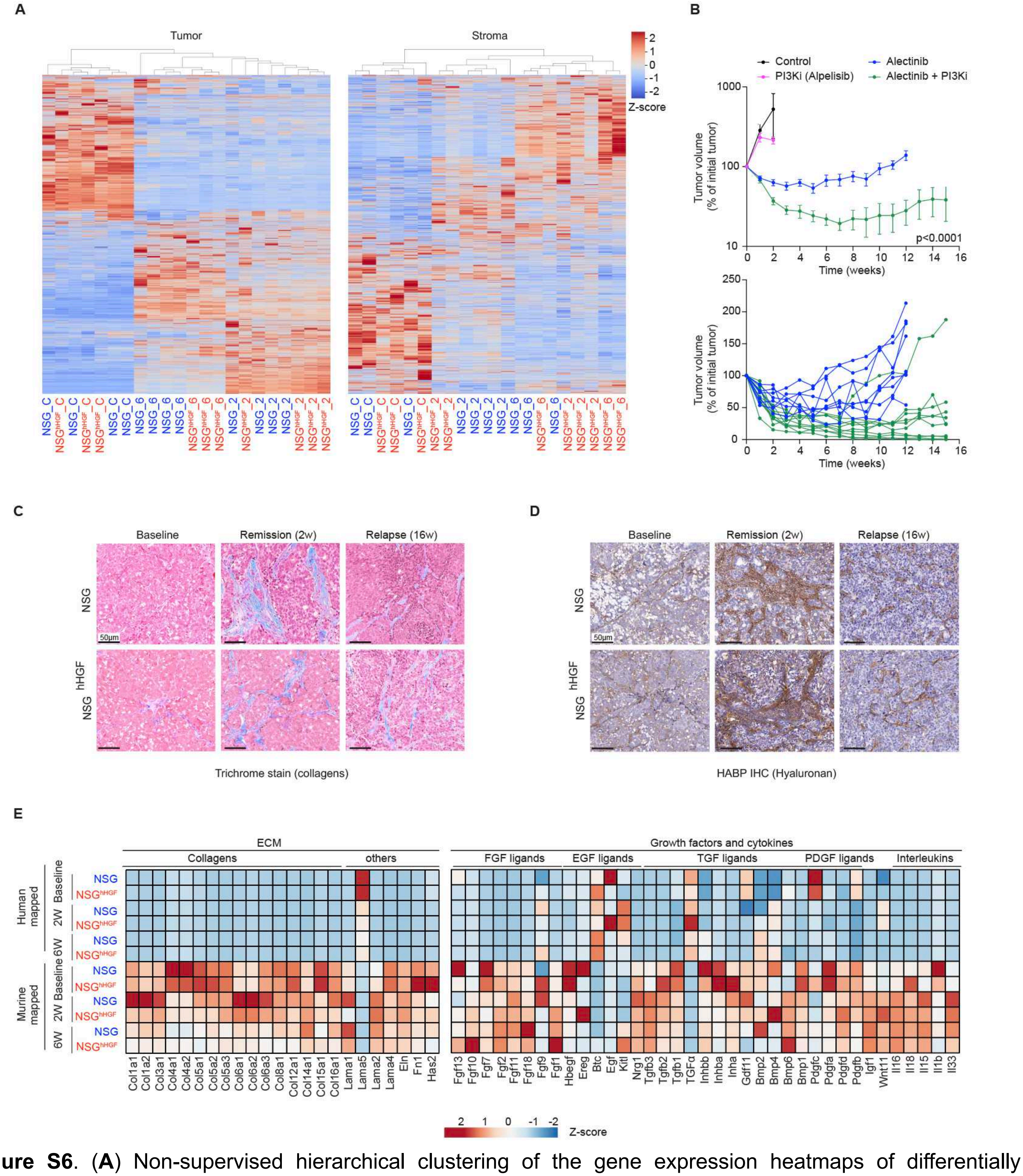

Figure S6. (**A**) Non-supervised hierarchical clustering of the gene expression heatmaps of differentially expressed genes in the indicated samples. (**B**) Volumetric responses of average (upper panel) and individual tumor traces (lower panel) of H3122 xenograft tumors in NSG hosts treated with vehicle control (N=4), 25mg/kg alpelisib (N=6), 25 mg/kg alectinib (N=8) and alectinib/alpelisib combination (N=10). Error bars represent SEM. P values represents the significance of the interaction term of 2-way ANOVA analyses between the alectinib and alectinib/alpelisib combination treated group. (**C**) Representative images of Mason Trichrome staining of the indicated tumors. (**D**) Representative images of the HABP IHC staining of the indicated tumors. (**E**) Heatmaps of expression of the indicated genes in the indicated experimental tumors.

Figure S7

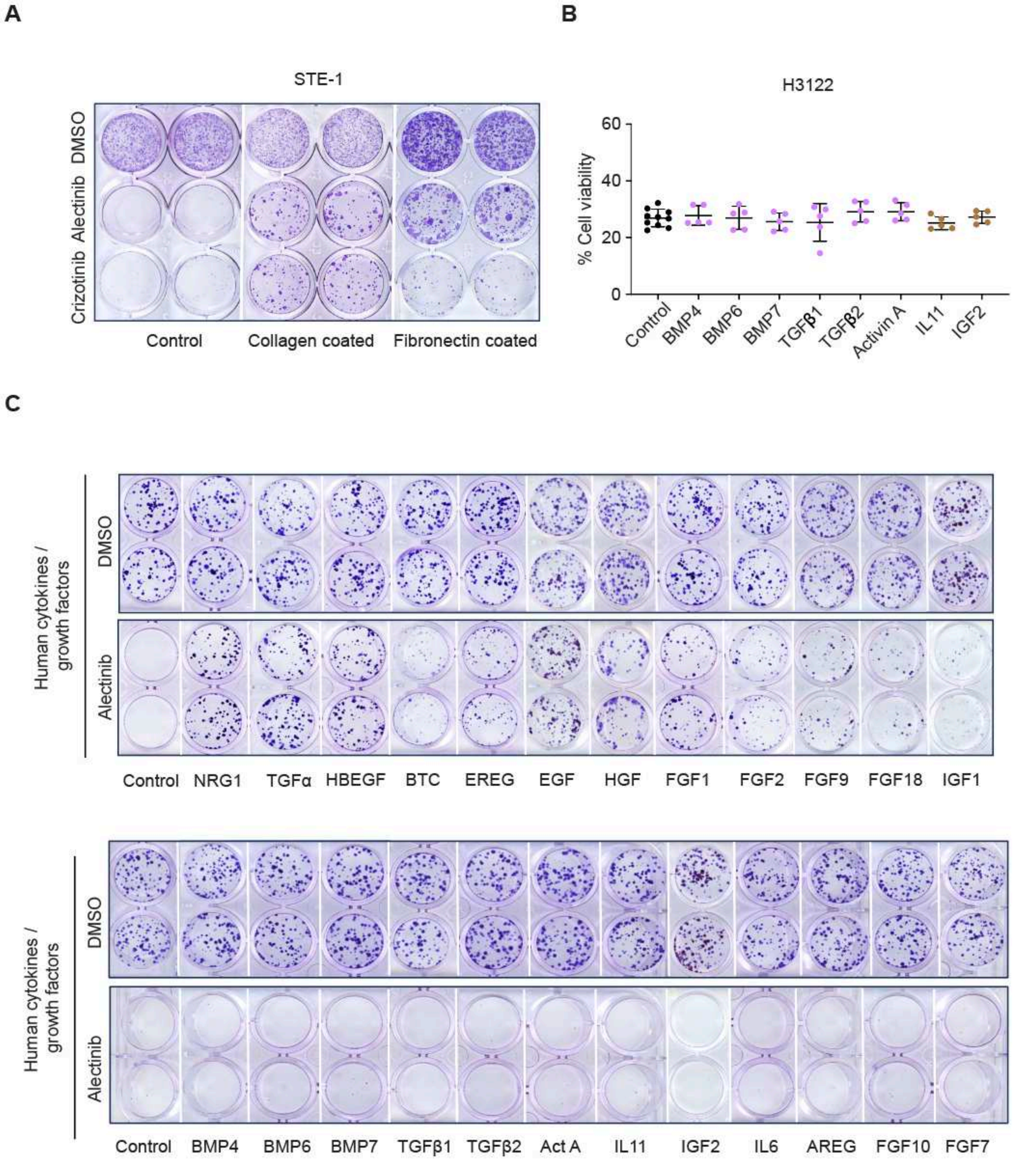

Figure S7. (**A**) Images of crystal violet staining of the clonogenic assay of STE1 cells cultured for 20 days in regular, collagen, or fibronectin-coated plates in the presence of DMSO control, 0.1 μM alectinib, and 0.5 μM crizotinib. (**B**) Cell viability assay for H3122 cells grown in the presence of the indicated factors (50 ng/ml). (**C**) Images of crystal violet staining of the 10 days culture of H3122 cells in the presence of the indicated growth factors (50 ng/ml).

Figure S8

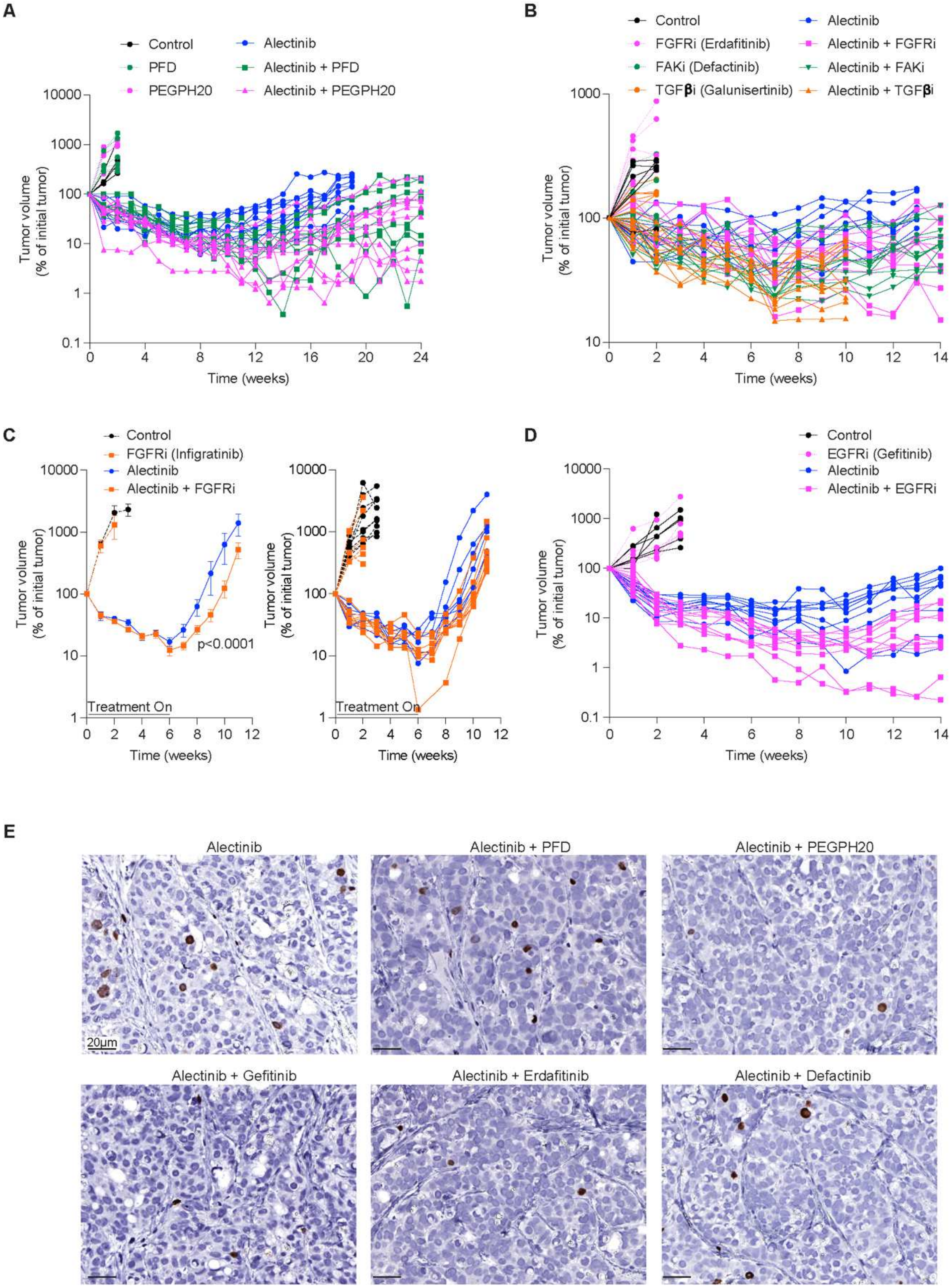

Figure S8. (**A**) Volumetric traces of individual H3122 xenograft tumors treated with vehicle control, 900 mg/kg pirfenidone, 0.1mg/kg PEGPH, 25 mg/kg alectinib, alectinib/pirfenidone and alectinib/PEGPH20 combinations. (**B**) Volumetric traces of individual H3122 xenograft tumors treated with vehicle control, 20 mg/kg erdafitinib, 50 mg/kg defactinib, 75 mg/kg galunisertib, 20 mg/kg alectinib and the indicated combinations. (**C**) Volumetric traces of averages (left) and individual tumors (right) of H3122 xenograft tumors treated with vehicle control (N=10), 30 mg/kg infigratinib (N=6), 20 mg/kg alectinib (N=6), and alectinib/infigratinib combination (N=8). All mice received treatment break post 6 weeks. Error bars represent SEM. (**D**) Volumetric traces of individual tumors of H3122 xenograft tumors treated with vehicle control, 40 mg/kg gefitinib, 20 mg/kg alectinib and alectinib/gefitinib combination. (**E**) Representative images of anti-BrdU IHC staining of tumors tissues from H3122 xenograft mice treated with the indicated therapies for 7 days.

Figure S9

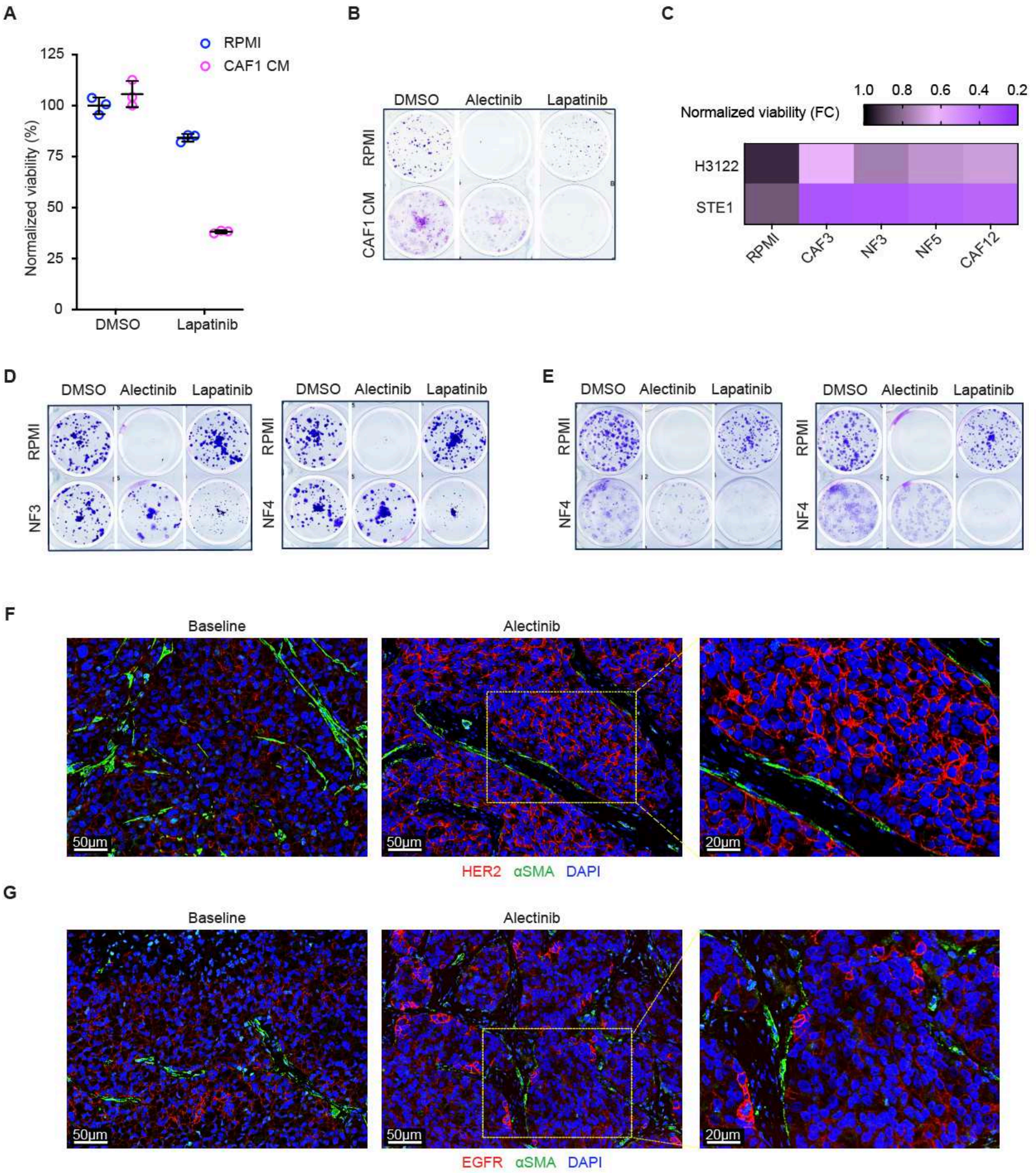

Figure S9. (**A**) Impact of CAF1 CM on sensitivity of STE-1 cells to 10 μM lapatinib in 4 days CellTiter-Glo assay. (**B**) Impact of CAF1 CM on sensitivity of STE-1cells to 0.1 μM alectinib and 10 μM lapatinib in 10 days Crystal Violet clonogenic assay. (**C**) Heatmap summary of the fibroblast CM induced lapatinib (10 μM) sensitization in H3122 and STE-1 cells. The sensitivity is presented as the fold change in cell viability as compared to the RMPI media control. (**D, E**) Crystal violet stain of the 10-day culture of H3122 (D) and STE-1 (E) cells in RPMI, or fibroblast CM in 0.1 μM alectinib, 10 μM lapatinib and DMSO control. (**F, G**) Representative images of IF staining of the indicated H3122 xenograft tumors with HER-2 (red) and αSMA (green) (F), or EGFR (red), and αSMA (green) (G).

Figure S10

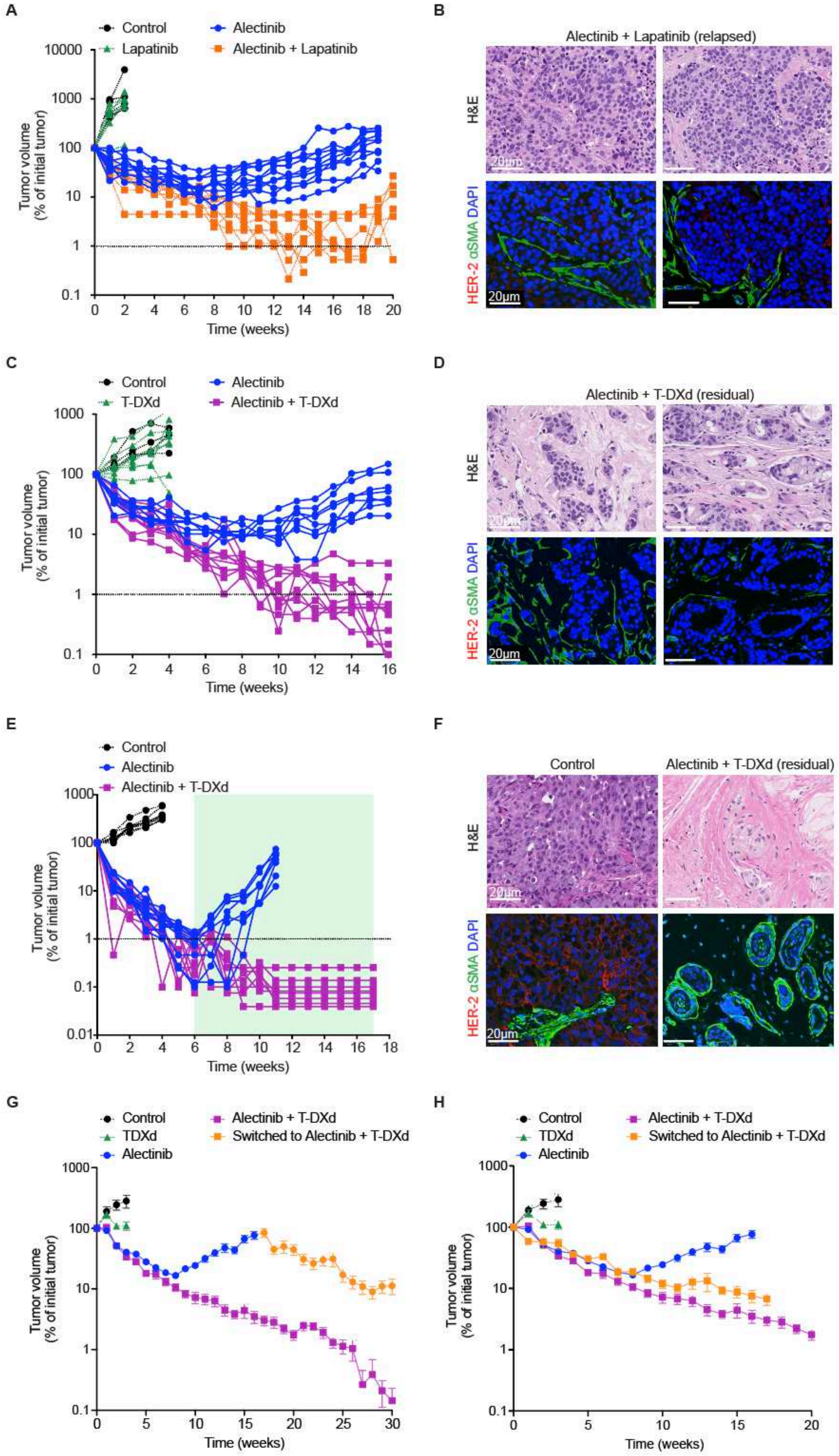

A
Control
Lapatinib
Alectinib
Alectinib + Lapatinib
Tumor volume (% of initial tumor)
Time (weeks)
B
Alectinib + Lapatinib (relapsed)
H&E
HER-2 αSMA DAPI
20μm
C
Control
T-DXd
Alectinib
Alectinib + T-DXd
Tumor volume (% of initial tumor)
Time (weeks)
D
Alectinib + T-DXd (residual)
H&E
HER-2 αSMA DAPI
20μm
E
Control
Alectinib
Alectinib + T-DXd
Tumor volume (% of initial tumor)
Time (weeks)
F
Control
Alectinib + T-DXd (residual)
H&E
HER-2 αSMA DAPI
20μm
G
Control
TDXd
Alectinib
Alectinib + T-DXd
Switched to Alectinib + T-DXd
Tumor volume (% of initial tumor)
Time (weeks)
H
Control
TDXd
Alectinib
Alectinib + T-DXd
Switched to Alectinib + T-DXd
Tumor volume (% of initial tumor)
Time (weeks)

Figure S10. (**A**) Volumetric traces of individual H3122 xenograft tumors treated with vehicle control, 100 mg/kg lapatinib, 25 mg/kg alectinib, and alectinib/lapatinib combination. (**B**) Representative images of the indicated endpoint H3122 xenograft tumors from (A) stained with H&E (upper panel) and IF co-staining against HER-2 (red) and αSMA (green) (lower panel). (**C**) Volumetric traces of individual H3122 xenograft tumors treated with vehicle control, 10 mg/kg T-DXd, 25 mg/kg alectinib, or alectinib/T-DXd combination. (**D**) Representative images of the indicated endpoint H3122 xenograft tumors stained with H&E (upper panel) and IF co-staining against HER-2 (red) and αSMA (green), DAPI (blue) (lower panel). (**E**) Volumetric traces of individual STE1 xenograft tumors treated with vehicle control, 20 mg/kg T-DXd, 12.5 mg/kg alectinib, and alectinib/T-DXd combination. (**F**) Representative images of the indicated endpoint STE1 xenograft tumors stained with H&E (upper panel) and IF and IF co-staining against HER-2 (red) and αSMA (green) (lower panel). Dotted lines in A, C and E indicate detectability threshold for volumetric measurements. (**G, H**) Volumetric traces of averages of H3122 xenograft tumors treated with vehicle control (N=8), 10 mg/kg T-DXd (N=8), 25 mg/kg alectinib (N=10), or alectinib/T-DXd combination (N=10). The impact of combination therapy on volumetric responses of tumor relapsed on alectinib monotherapy is shown in (G) and re-normalized at the point of switching to combination therapy in (H).
